# Bisphosphonates loaded nanoparticles in microparticles: a potential macrophage targeting and repolarizing drug delivery system

**DOI:** 10.1007/s13346-025-01889-7

**Published:** 2025-06-06

**Authors:** Paul N. K. Sagoe, Benjamin Zink, Era Jain

**Affiliations:** 1 Department of Biomedical and Chemical Engineering, Syracuse University, Syracuse, NY 13244 USA; 2 Bioinspired Syracuse: Institute for Material and Living System, Syracuse University, Syracuse, NY 13244 USA; 3 SUNY Upstate Medical University, Syracuse, NY 13210 USA

**Keywords:** Bisphosphonate, M1/M2 macrophage repolarization, Immunomodulation, NF-κB and ROS scavenging, Drug delivery, Nanoparticles in Microparticles

## Abstract

Bisphosphonates (BPs) are widely used in treating bone-related conditions, with emerging evidence supporting their potential in treating both skeletal and extra-skeletal diseases. However, their clinical utility is limited by high cytotoxicity, particularly toward macrophages, leading to immune system disruption upon frequent use. This limitation highlights the need for an effective drug delivery system. While nanoparticle formulations improve pharmacokinetics and biodistribution, they often suffer from limited drug loading capacity, poor sustained-release ability, and increased cytotoxicity due to their rapid and excessive intracellular uptake.

Here, we present a multifunctional formulation, composed of calcium-zoledronic acid nanoparticles (CaZol NP) encapsulated within polymeric microparticles (CaZol NiM), designed to address many challenges associated with therapeutic use of zoledronic acid (Zol). CaZol NiM improves cellular uptake of Zol, facilitates pH sensitive sustained release of Zol and allows ligand mediated uptake by macrophages. The controlled release of Zol from CaZol NiM effectively reduces Zol’s cytotoxic effects on macrophages, enabling their immune modulation by suppressing NF-κB and reactive oxygen species (ROS) activity, while promoting macrophage repolarization from their pro-inflammatory M1 state.

Altogether, these findings highlight the potential of CaZol NiM in minimizing off-target effect and expand the clinical applications of Zol in managing both skeletal and extra-skeletal inflammatory disorders.

**Graphical Abstract. Image created using Biorender.com:**

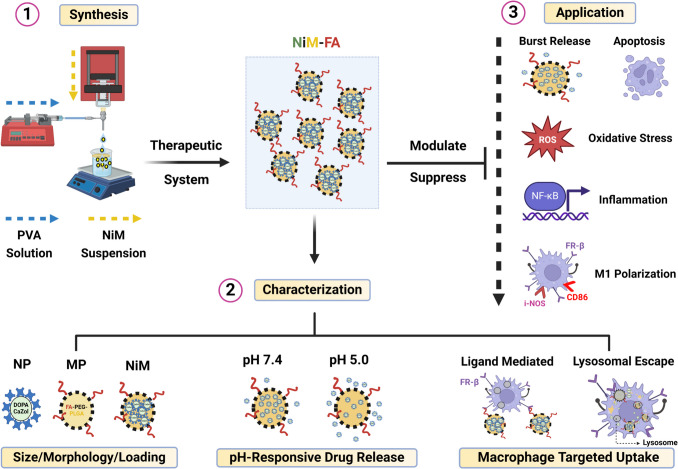

**Supplementary Information:**

The online version contains supplementary material available at 10.1007/s13346-025-01889-7.

## Introduction

Bisphosphonates (BP) are acknowledged as first-line, cost-effective drugs with significant clinical potential for the treatment of diverse inflammatory, cancer, and bone-related diseases, including osteoarthritis, osteoporosis, and cancer bone metastasis [1–8]. This recognition stems from their high affinity and toxicity towards monocyte derivatives, particularly osteoclasts and macrophages, which have been identified as significant contributors to these diseases. To date, numerous bisphosphonates (BPs) have been developed, with the third generation being the most potent. Among these, zoledronic acid (Zol), a third-generation nitrogen-containing BP, has garnered significant attention for its high therapeutic potency, chondroprotective properties, and immunomodulatory effects, particularly on macrophages. Additionally, Zol has shown widespread applicability in treating both skeletal and non-skeletal disorders in several preclinical studies [9–15].

However, the ultimate realization of the clinical application of Zol remains limited owing to poor pharmacokinetic properties, as depicted by its inherent hydrophilic and anionic nature. As such, Zol upon delivery suffers the fate of early clearance, low drug bioavailability, high cytotoxicity and inefficient cellular and tissue targeting [1, 16]. Consequently, achieving therapeutic efficacy requires repeated delivery of high dosage [17], which substantially heightens the risk of adverse outcomes, including kidney failure, the potential for systemic toxicity, and the occurrence of bisphosphonate-related osteonecrosis of the jaw (BRONJ) [10, 17–20]. Considering these challenges, it is imperative to minimize adverse side effects while maximizing the therapeutic potential of Zol. This approach is critical for advancing its current clinical application, enabling repurposing opportunities for non-skeletal disorders, and ultimately improving therapeutic outcomes.

Efforts towards encapsulating Zol into delivery systems such as liposomes, dendrimers, micelles, nanoparticles, and microparticles have therefore emerged as a promising strategy for achieving therapeutic efficacy of this potent yet vulnerable drug [15]. For instance, previous studies utilizing liposomes for the delivery of Zol demonstrated enhanced pharmacokinetics and improved biodistribution in the target tissue [21, 22]. However, it is noteworthy that the encapsulation efficiency of these liposomes containing Zol was relatively low. Additionally, these Zol-encapsulated liposomes were reported to exhibit high systemic toxicity in vivo in mice compared to free Zol [22].

Moreover, recent research has demonstrated that complexing Zol with metal composites, specifically calcium, enables the synthesis of CaZol nano-complexes, which hold promise for overcoming key limitations of current formulations [7, 23]. Unlike liposomes, these nano-complexes offer improved encapsulation efficiency, reduced systemic toxicity, and significantly enhanced drug bioavailability. Additionally, their pH sensitivity also facilitates lysosomal escape during cellular uptake, promoting efficient release of the therapeutic payload within the cell [5, 7, 23–25].

However, CaZol nano-complexes exhibit high burst release, severely limiting their clinical utility for skeletal and non-skeletal disorders [5, 23, 24]. Notably, by incorporating CaZol nano-complexes into nanoparticles made of poly(lactic-co-glycolic) acid (PLGA) polymer, [26], Li et al. observed a significant reduction in release of Zol from ~ 35% to ~ 5% in 48 h [23]. This interesting finding underscores the potential of an external carrier as a promising system for minimizing the burst release of Zol from CaZol nano-complexes.

While nanoparticle formulations are advantageous for targeted delivery, their rapid and excessive intracellular uptake often leads to increased cytotoxicity compared to free Zol, particularly toward macrophages. Thus, with macrophages being key players in maintaining homeostatic balance, long-term use of these CaZol-loaded PLGA NP could potentially cause several side effects. Moreover, although the distinct small size of nanoparticles facilitates passive targeting and infiltration into target sites, it also makes them highly susceptible to clearance from circulation through vascular and lymphatic drainage [27, 28]. Additionally, even with surface modifications with polyethylene glycol (PEG) or distinct targeting ligands, effectively preventing non-specific targeting towards neighboring tissues remains a considerable challenge. This is primarily because the intrinsic small size of nanoparticles makes them highly susceptible to transportation by circulating body and tissue fluids, leading to unintentional elimination and redirection to non-target sites [29–33]. These, among other reasons, make nanoparticles less suitable as external cargo for sustained release application despite their wide range benefits and potential for targeted drug delivery.

Notably, microparticles (MP), given their larger size, offer numerous advantages that make them suitable as carriers for sustained drug delivery applications. These advantages encompass their capacity to tune drug release, load sufficient drug payloads; ultimately enhancing drug loading and biodistribution. Additionally, their potential to exhibit prolong retention time as they are not susceptible to rapid clearance during circulation makes them preferred in clinical application where repeated delivery such as injection isn’t desirable [34–36]. Thus, to enhance the application of microparticle systems while retaining the benefits of nanoparticles, recent studies highlight that multifunctional systems incorporating nanoparticles within microparticles offer precise control over drug release and distribution, enabling targeted and sustained delivery to disease-specific tissues [37–39].

Here to take advantage of the robustness of polymeric microparticles, combined with the unique characteristics of CaZol nano-complexes, we present a multifunctional drug delivery system comprising CaZol NP encapsulated within PEG-PLGA microparticles to form CaZol NiM. Our results demonstrate that CaZol NiM minimizes the initial burst release of Zol at physiological pH while enabling pH-sensitive, rapid drug release under acidic conditions. Notably, introducing folic acid (FA) as a ligand enhanced the system’s ability to target macrophages, which upon uptake colocalized with lysosomes allowing for efficient pH-triggered intracellular release of Zol.

The targeted system, CaZol NiM-FA, showed strong anti-inflammatory and immunomodulatory properties by lowering NF-κB expression, suppressing ROS production, and repolarizing pro-inflammatory macrophage towards a non-inflammatory state.

Unlike free Zol or Zol-loaded nanoparticles (CaZol NP), the CaZol NiM-FA formulation preserved high cell viability, demonstrating its capacity to reduce Zol-related cytotoxicity effectively. By leveraging the natural affinity of bisphosphonates like Zol for macrophages [11, 24], CaZol NiM-FA offers a robust, targeted and sustained-release platform with significant potential for expanding the clinical applications of bisphosphonates in managing macrophage mediated inflammatory responses in skeletal and extra-skeletal disorders.

## Materials and methods

### Materials

Poly (ethylene glycol)-methyl ether-block -poly (lactide-co-glycolide) (PEG-PLGA_50:50_, M_n_ PEG of 2000 Da and M_n_ PLGA of 10,000 Da), folate-poly (ethylene glycol)-b-poly(lactide-co-glycolide), TritonX-100, and coumarin 6 dye were purchased from Sigma Aldrich (St. Louis, MO, USA). 1,2-dioleoyl-sn-glycero-3-phosphate monosodium (DOPA) was purchased from Avanti Polar Lipid, Inc. (Alabaster, AL). Zoledronic acid monohydrate, Igepal CO-520, Rhodamine 6G dye, 3-(4, 5 dimethylthiazol-2-yl)-2, 5-diphenyltetrazolium bromide(MTT), cyclohexane, chloroform, 1-hexanol, polyvinyl alcohol (PVA) (87–89% hydrolyzed, high molecular weight), phosphate buffer saline (PBS), 4-(2-hydroxyethyl)piperazine-1-ethanesulfonic acid (HEPES) buffer, polyvinyl chloride (PVC) tubing, syringes (glass and plastic), and other reagents were obtained from Fisher Scientific (Waltham, MA, USA) All other chemicals and reagents used in this study were of analytical grade.

### Preparation and characterization of DOPA-coated calcium zoledronate nanoparticles (CaZol NP)

#### Preparation of DOPA-coated calcium zoledronate nanoparticles (CaZol NP)

**Scheme 1:**
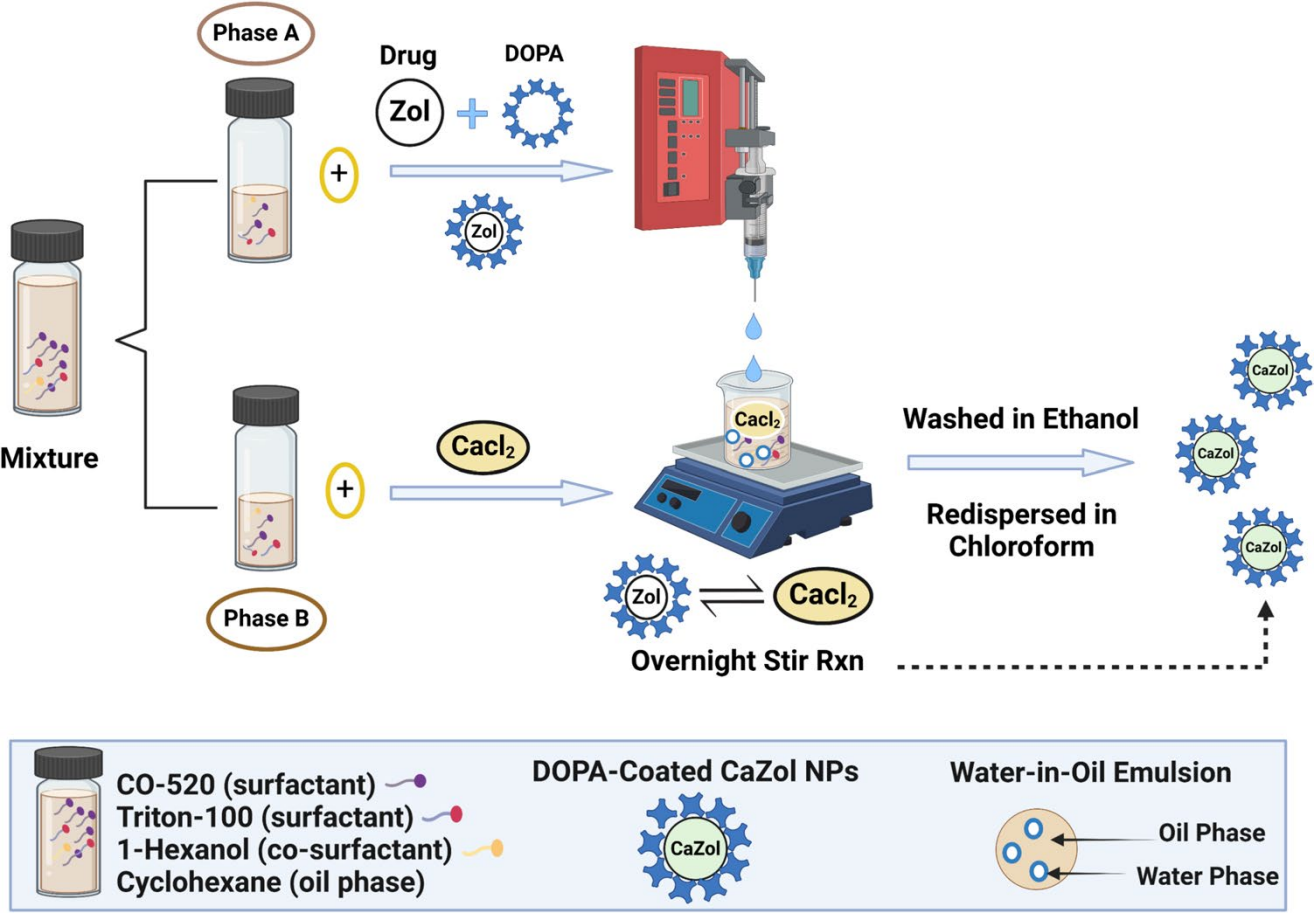
Synthesis of CaZol nanoparticles via reverse-emulsification. Image created using Biorender.com

CaZol NPs, coated with the hydrophobic anionic lipid DOPA, were synthesized using a previously described reverse microemulsion technique [25]. As illustrated in Scheme 1, 10 mL of water-in- oil (w/o) solution was prepared by combining CO-520 (surfactant), Triton X-100 (surfactant), 1-hexanol (co-surfactant), and cyclohexane (oil phase) at a volumetric ratio of 10.5/7.5/5/77, v/v/v/v, divided into two halves of 5 mL each.

To form the Zol phase 100 μL of Zol solution (25 mM, in 0.5 M Tris buffer, pH 10) and 133 μL of DOPA solution (4 mg/200 μL in chloroform) were added dropwise to one of the separated halves. In the other half, 100 μL of CaCl_2_ solution (500 mM in 0.5 M Tris buffer, pH 10) was added to form a calcium phase. Following this, the Zol phase was placed in a glass syringe and injected slowly at a rate of 10 mL/hr into the calcium phase under a magnetic stir rate of 600 rpm. After injection, the resulting mixture was allowed to further stir overnight for complete complexation reaction between Zol with Ca, leading to formation of DOPA coated CaZol NP. To precipitate and purify the nanoparticles, an equal volume of ethanol was added to the emulsion, and the mixture was subjected to a high-speed centrifugation at (12,500 × g for 30 min). The procedure was repeated twice. The purified nanoparticles were redispersed in chloroform and sonicated in a water bath for 30 min to obtain even dispersion. Finally, the nanoparticles were filtered through a 0.2 μm PTFE syringe filter, and the resulting suspension in chloroform was either stored at -20 °C for future studies or immediately characterized.

#### Characterization of DOPA-coated calcium zoledronate nanoparticles (CaZol NP)

Morphological analysis of CaZol nanoparticles (NPs) was performed using transmission electron microscopy (TEM) with a JEOL JEM-1400 series 120 kV instrument. Briefly, samples were prepared by dropping a small amount of NP suspension (3 μL) onto carbon coated copper grids. Following chloroform evaporation, the dried grids were placed on the sample stage for imaging.

The number-average diameter (Dn) of CaZol NP was calculated by measuring at least 120 individual particles from representative TEM images using ImageJ software. Additionally, the hydrodynamic size (Dh) of NP, polydispersity index (PDI), zeta potential, and particles concentration were determined by dynamic light scattering (DLS) technique using the Zetasizer Ultra (Malvern instrument). Briefly, 1 mL of CaZol NP suspension in chloroform at 1:100 dilution was gently injected into an appropriate glass cuvette cell (PCS8501, Malvern Instruments, MA) for size and particle concentration measurement. Measurements were taken at 25 °C with a material refractive index of 1.45, and a dispersant viscosity of 0.536 mPa·s. for chloroform.

For zeta potential measurements, the CaZol NP suspension was placed in a glass vial, and after chloroform evaporation, the residual nanoparticles were resuspended in DI water. The suspension was then transferred to a disposable plastic capillary cell (DTS1070, Malvern Instruments) and measured at 25 °C with a material refractive index of 1.45 and a dispersant viscosity of 0.8872 mPa·s for water. All measurements were repeated in triplicate, and average values were reported.

To study the stability of nanoparticles, equivalent concentrations of CaZol NPs were separately placed in chloroform, PBS (7.4 pH), and PBS (5.0 pH) at room temperature for 7 days, and the subsequent degree of particles degradation was qualitatively observed via TEM.

To fluorescently label CaZol NP, 5 mg/mL coumarin-6 dye was dissolved in ethanol, and added to 2 mg/mL of CaZol NP suspension in chloroform at 1%v/v. The mixture was allowed to stir overnight to allow complete evaporation of chloroform. The resulting thin film was then hydrated with 1 mL deionized water, and the recovered CaZol NPs were passed through a 0.2 μm syringe filter to obtain coumarin-6 labeled DOPA-CaZol NPs.

### Preparation and characterization of blank microparticles (PEG-PLGA MP), and DOPA-coated CaZol nanoparticles loaded into microparticles (CaZol NiM)

#### Preparation of blank microparticles and nanoparticles in microparticles (CaZol NiM)

Polymeric microparticles made of PEG-PLGA were fabricated following our previously reported phase separation method based on coaxial needle technology with minor modifications [40]. Briefly, 500 µL of the organic phase consisting of 0.1% w/v PEG-PLGA in dichloromethane (DCM) was simultaneously injected along with 20 mL of the aqueous phase consisting of 5% w/v polyvinyl alcohol (PVA) at flow rates of 1 mL/hr and 40 mL/hr, respectively via a coaxial needle into a clean glass beaker or PTFE collecting dish, maintained under constant stirring (1500 RPM). Following the injection of the emulsions and complete evaporation of the DCM, the synthesized microparticles were collected and washed with deionized water via centrifugation (1500 RPM for 5 min) to remove residual PVA. The pelleted microparticles hereafter referred to as blank microparticles (PEG-PLGA MP), were redispersed in deionized water or lyophilized and stored for future studies.

**Scheme 2:**
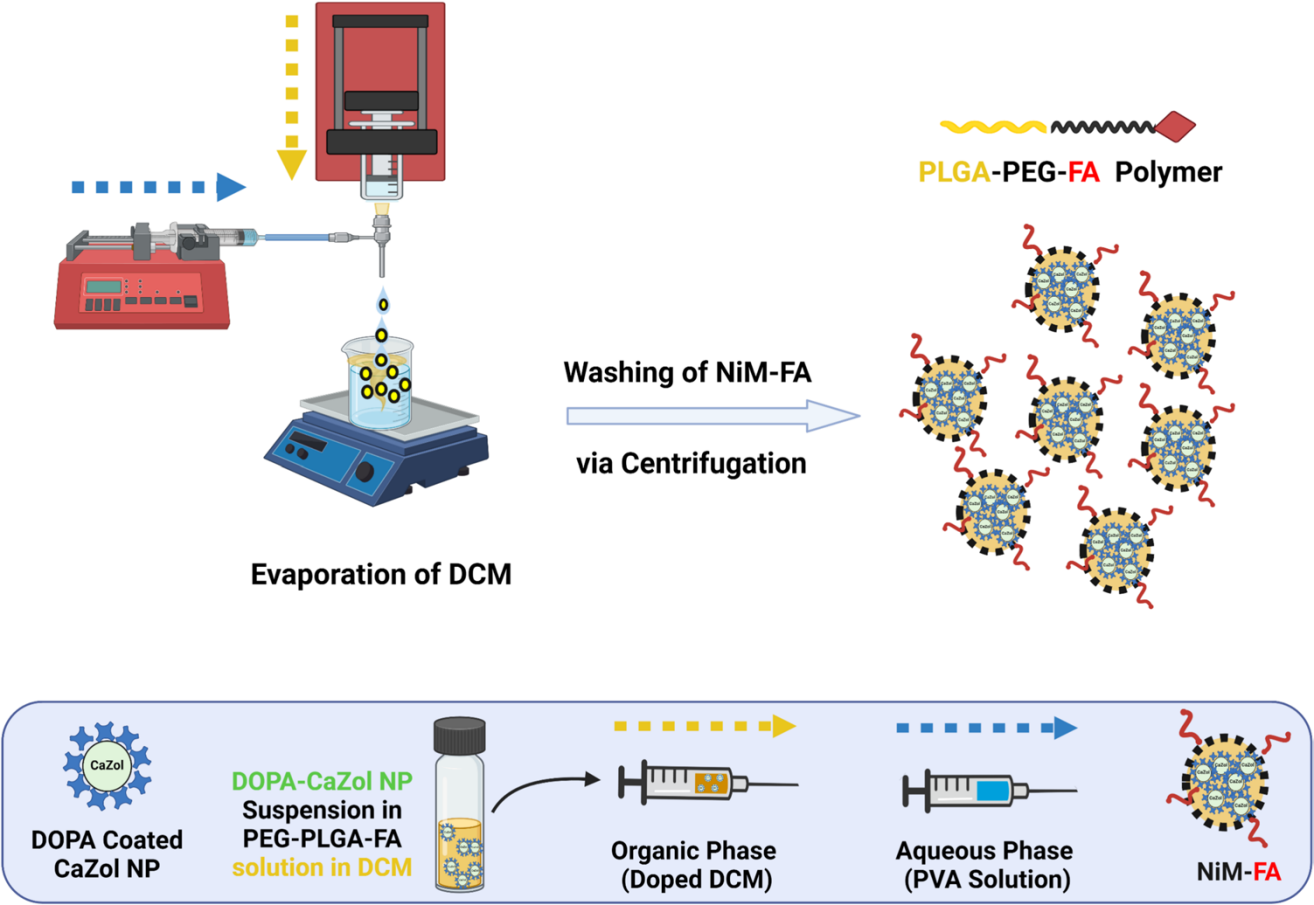
Synthesis of CaZol-NiM by loading nanoparticles in microparticles via coaxial-flow phase separation technique. Image created using Biorender.com

To load nanoparticles into microparticles to form (CaZol NiM), we followed the procedure described in Scheme 2. Briefly, 100 µL of CaZol NP suspension in chloroform estimated at 100 μg/mL of Zol with a known particle concentration were dispersed in a glass vial and allowed for chloroform evaporation overnight. Following this, 500 µL of the organic phase consisting of 0.1% w/v PEG-PLGA in DCM was added to the glass vial to resuspend the CaZol NP residue, forming a doped organic phase. Then nanoparticles in microparticles were synthesized, purified, and stored following the same process described for the synthesis of PEG-PLGA microparticles.

#### Characterization of blank microparticles and nanoparticles in microparticles (CaZol NiM)

To characterize the size of blank microparticles and CaZol NiM, an inverted microscope was used to capture brightfield images of the particles in suspension from a glass slide, and Image J software was used to subsequently measure the diameter of the captured particles. To determine the morphology of the particles, the lyophilized form of blank microparticles and CaZol NiM were independently sputter coated, and their microscopic images were visualized using scanning electron microscopy (SEM; JEOL JSM-IT100).

CaZol NiM were further characterized using fluorescent microscopy, Fourier transforms infrared (FTIR, Thermo Nicolet™ Spectrometer iS5), and energy-dispersive X-ray (EDAX, JEOL JSM-IT100) to confirm CaZol NP encapsulation the microparticles. For fluorescent microscopy characterization, Zol NP were labeled with DiD dye (1% v/v of 5 mg/mL DiD in ethanol added to CaZol NP suspension in chloroform). After allowing the chloroform to evaporate, the DiD labelled CaZol NPs were collected, centrifuged and filtered as described above. The organic phase used in the fabrication of microparticles were also labeled with coumarin6 dye (1%v/v of 5 mg/mL) by mixing the dye in the organic phase. Subsequently the coum-6 labelled organic phase was used to resuspend the DiD-labeled CaZol NP to obtain a fluorescently doped organic phase for CaZol NiM. Coum-6 labelled organic phase without DiD-labelled CaZol NP mixture was used to synthesize CaZol NiM to serve as a control, and fluorescent images of the respective channels were captured with confocal microscope.

For FTIR analysis, free Zol, CaZol NP and CaZol NiM were synthesized and characterized for the presence of phosphate and C-N stretching bands, both being distinct functional groups distinct to Zol. As a control, blank microparticles (PEG-PLGA MP), without Zol loading, were similarly investigated. Additionally, using EDAX analysis, the presence of calcium and phosphorus was validated as evidence of CaZol NP encapsulation in microparticles to form CaZol NiM. Blank microparticles were considered control. For the synthesis of folate conjugated NiM (NiM-FA), PEG-PLGA polymer was mixed with folic acid conjugated PEG-PLGA polymer (PEG-PLGA-FA) at a ratio of 5:1 w/w and used for NiM fabrication at a concentration of 1% w/v as described above. PEG-PLGA-FA microparticles were characterized by FTIR to confirm the successful incorporation of folic acid ligand.

### Determination of drug loading, encapsulation efficiency, and loading capacity of nanoparticles in microparticles

To determine the number of nanoparticles encapsulated per microparticle, three representative fluorescent images of CaZol NiM were first selected. From each image, at least 30 individual microparticles were analyzed. For each selected microparticle, the number of encapsulated nanoparticles (CaZol NP), identified through DiD staining (DiD-CaZol NP), was counted from the coumarin-6 stained microparticles. The total number of nanoparticles counted for all selected microparticles within each image was then divided by the total number of microparticles analyzed in that image. Finally, the mean values calculated from the three representative images were averaged to determine the overall number of nanoparticles encapsulated per microparticle and expressed as mean ± SD. Blank microparticles stained with DiD or Coumarin-6 dyes but without nanoparticles loading were synthesized to serve as control.

The encapsulation efficiency (%) and percent drug loading (w/w) of Zol in CaZol NPs and CaZol NiM were determined spectrophotometrically, following a previously described procedure with minor modifications [5, 41]. Briefly, particles were added to pre-weighed microcentrifuge tubes and the weight of particles was determined by subtracting the weight of the empty tube. The particles with known weight were digested overnight in 1 mL of 0.1 M HCl (0.005 M PBS). followed by determination of Zol concentration using a UV–VIS spectrophotometer (Evolution 60) by recording the absorbance at 215 nm. The encapsulation efficiency (%) and percent drug loading (w/w) of Zol were determined using the formulas below:

$${{E}}{{E}}{\%}=\frac{{{{M}}}_{{{E}}{{n}}{{c}}}}{{{{M}}}_{{{I}}{{n}}{{i}}{{t}}}}\times 100$$

$${{D}}{{L}}{\%}=\frac{{{{M}}}_{{{E}}{{n}}{{c}}}}{{{{M}}}_{{{D}}{{s}}}}\times 100$$

where M_Enc_ is the mass of encapsulated Zol, M_Init_ is the initial mass of Zol used for the formulation, and M_Ds_ total mass of the particles (CaZol NP or CaZol NiM). Further, relative efficiency of encapsulating CaZol NP or free Zol in microparticles was also determined by loading equivalent concentration of Zol loaded into PEG-PLGA to form CaZol NiM and PEG-PLGA-Zol MP respectively and comparing corresponding encapsulation efficiencies as described above.

### In vitro release of Zol from CaZol NPs and CaZol NiM

CaZol NPs and CaZol NiM, containing equivalent amount of Zol, were resuspended in 1 mL of release medium consisting of 0.005 M PBS buffer of either pH 7.4 or 5.0 in 2 mL microcentrifuge tubes. The tubes were placed in a shaker incubator (C24-New Brunswick Scientific) and maintained at 37 °C and 300 RPM for release kinetics determination. At specific time intervals, the tubes were removed, centrifuged and 1 mL of the release medium was collected. An equal amount of fresh buffer was added to each to maintain sink conditions. The concentration of Zol in the aliquots collected at each time point was determined spectrophotometrically as described above using 0.005 M PBS as blank.

### In vitro cellular assays

Murine RAW264.7 macrophages (American Type Culture Collection (ATCC)) were cultured in RPMI 1640 medium supplemented with 10% FBS (Gibco, NY, USA) and 1% penicillin–streptomycin (PS) at 37 °C in a humidified atmosphere containing 5% CO_2_. Cells were passaged when 80% confluent by detaching using trypsin–EDTA, centrifuging to get a cell pellet, resuspending in fresh media and seeding in a fresh tissue culture-treated-flask. All studies were conducted using cells with passage number 8 or less.

#### Activation of RAW 264.7 macrophages

RAW 264.7 cells were seeded at the required density and incubated overnight. Except for macrophage phenotype repolarization studies, all cellular studies were conducted by first activating the macrophages to M1 state by treating with lipopolysaccharides (LPS, 1 μg/mL) for 4 h and washing thrice with PBS buffer (pH 7.4) before further treatment according to the desired experiment. Non-LPS activated macrophages were used as controls where necessary.

#### Cellular uptake and intracellular localization of CaZol NiM

RAW 264.7 cells were seeded at a density of 3 × 10^5^ cells in an 8-chamber ibidi μ-slides and activated as described. Post washing macrophages were incubated for 4 h with Coum-6 labeled particles (CaZol NPs or CaZol NiM) resuspended in RPMI 1640 cell culture medium.

To study ligand mediated uptake activated RAW 264.7 cells were incubated with Coum-6 labeled CaZol NiM- FA. Additionally, cells pre-treated with 1 mM of free folic acid for 1 h post LPS activation were used as controls. After the incubation, the cells were washed three times with PBS (pH 7.4), fixed in 4% paraformaldehyde (PFA), and stained with Phalloidin-iFluor 532 (1:1000, ab176755, Abcam) 1 h at room temperature and washed thrice with PBS.

To determine the intracellular localization cells were stained with Lysotracker Red DND-99 (50 nM), fixed in 4% PFA for 15 min, and washed with PBS buffer.

Post washing all the immunostained samples were counterstained with DAPI (1 µg/mL) for 20 min, washed thrice in PBS and imaged using the Zeiss LSM 980 with Airyscan 2 or VT-iSIM super resolution spinning disk confocal microscope.

For quantitative analysis of cellular uptake of particles, RAW 264.7 cells were seeded overnight at a density of 2 × 10^5^ cells per well onto 6-well plates and activated with LPS as described. Activated RAW 264.7 cells were incubated with Coum-6 labeled particles (CaZol NPs or CaZol NiM; with equivalent concentration of Coum-6) for 4 h and harvested. Cells were washed with PBS thrice. Cellular uptake was measured by determining the fluorescent intensity under the FITC channel using BD Accuri C6 flow cytometer and counting 10,000 events in the population. Data was analyzed using FlowJo software.

#### In vitro cytotoxicity of zoledronic acid and CaZol NiM

Activated RAW 264.7 macrophages (96-well plates; cell density of 5 × 10^3^ cells/well; LPS (1 μg/mL for 4 h) were treated with free Zol added to serum free media at different concentrations (1, 5, 25, 50, and 100 μg/mL) for 3, 12, and 24 h. Untreated cells were used as control. After the desired treatment times, 10 μL of MTT solution (5 mg/mL) in PBS was added to each well, incubated for 4 h in CO_2_ incubator at 37 °C. After incubation, the medium was discarded, 150 μL DMSO was added, and the plates were gently shaken for 10 min for the complete dissolution of formazan crystals and the absorbance (abs) was measured at 570 nm using a microplate reader (Biotek Synergy 2). The relative cell viability was calculated using the formula below.

$$Cell\ Viability\%=\frac{{ (Abs) W}_{treated {- (Abs) W}_{blank} }}{{ (Abs) W}_{control{- \left(Abs\right) W}_{blank} }}\times 100$$

where *W*_*treated*_ are cells treated with different zoledronic acid doses, *W*_*control*_ is untreated cells, and *W*_*blank*_ is media only samples.

To determine the cytotoxicity of Zol loaded particles, activated RAW 264.7 macrophages were incubated for 24 h with Zol-loaded particles (CaZol NP, CaZol NiM-FA) each having an equivalent Zol dose of 1, 5, 25, and 50 µg/mL. The cytotoxicity of these formulations was determined using MTT assay as described. Similarly, non-targeted CaZol NiM with equivalent Zol concentration of 25 ug/mL was synthesized and the degree of cytotoxicity was compared to the targeted CaZol NiM-FA by MTT at 24 h time point as an indirect assessment of both biocompatibility and targetability.

#### In vitro cellular apoptosis assay

For apoptosis studies, RAW264.7 (6-well plate; cell density of 5 × 10^5^ cells/well; LPS (1 μg/mL) 4 h) were incubated for 24 h with free Zol, CaZol NP, and CaZol NiM-FA each having an equivalent Zol dose of 25 µg/mL. Untreated cells and cells treated with blank PEG-PLGA MP-FA were used as controls. Following treatment, cells were harvested, washed with PBS twice, and stained with Annexin V-FITC Apoptosis Detection Kit (Thermo Fisher Scientific) as per the manufacturer's instructions. Samples were analyzed using BD Accuri C6 Plus flow cytometer by counting 20,000 events, and the data was analyzed using FlowJo software.

#### Detection of NF-κB activation

Activated RAW 264.7 macrophages (8-chamber ibidi μ-slides; cell density 3 × 10^5^ cells/well) were treated with CaZol NiM-FA (or CaZol NP and free Zol) at Zol dose equivalent of 25 µg/mL for 4 and 24 h. Untreated cells were used as controls. The cells were washed to remove any floating particles, fixed in 4% PFA (20 min), permeabilized with 0.1% Triton-X (10 min), washed twice with PBS, and blocked using 2% BSA (45 min). Cells were then stained with NF-κB p65 (F-6) Alexa Fluor® 488 (Santa Cruz, catalog # sc-8008 AF488) at 1:200 dilution in blocking buffer for 90 min. After washing with PBS three times, the cells were counterstained with DAPI (1 μg/mL), washed and imaged via confocal microscopy (Zeiss LSM 980). NF-κB activation was qualitatively observed by co-staining of the nucleus with DAPI and NF-κB p65, indicating nuclear translocation and activation.

For quantitative measurement of NF-κB nuclear translocation and activation, untreated RAW 264.7 cells (control) and cells treated with free Zol and Zol-particles (CaZol NP, CaZol NiM-FA) were stained for with NF-κB p65 (F-6) Alexa Fluor® 488 (2 µg/10^6^ cells in blocking buffer). After washing with PBS thrice, the cells were stained with 100 µL of FxCycle™ PI/RNase Staining Solution, for 15–30 min at room temperature in dark. The cells are then analyzed using the flow cytometer (BD Accuri, C6). Appropriate fluorescence minus one (FMO) and isotypes (IgG Alexa Fluor® 488) controls were included in this study to gate for the population of interest.

#### Detection of intracellular reactive oxygen species (ROS)

The production of intracellular ROS was assessed in activated RAW 264.7 cells using the 2,7-dichlorofluorescein diacetate (DCFH-DA) assay kit (Abcam). DCFH-DA is a non-fluorescent cell permeable probe which upon oxidation by ROS converts to DCF that can be fluorescently detected. The assay was monitored qualitatively and quantitatively by fluorescence microscopy and flow cytometry respectively. The cells were cultured and activated as described earlier and treated with CaZol NiM-FA particles for 4 and 24 h. Following treatment, the cells were washed twice with PBS, stained with 10 μM DCFH-DA solution in serum-free medium for 45 min, washed again using serum free media thrice, and counterstained with DAPI (1 µg/mL; 10 min). Samples were imaged using a fluorescent microscope (Leica DMI 6000). For flow cytometry, the cells were harvested after staining for 20 min with 10 μM DCFH-DA, washed, pelleted via centrifugation, and suspended in 500 µL of PBS and analyzed using a flow cytometer (10,000 events/sample). Unstained cells were included as controls for autofluorescence generated due to LPS activation.

#### Polarization of macrophages and assessment of phenotype repolarization by NiM-FA

RAW 264.7 macrophage cells were polarized into either the M1 or M2 state using cytokines specific to each phenotype. In brief, cells were stimulated for 24 h with E. coli lipopolysaccharide (LPS, 100 ng/mL, Thermo Fisher) and interferon-gamma (IFN-γ, 10 ng/mL, Peprotech) to induce M1 phenotypes or with 10 ng/mL each of interleukin-4 and 13 (IL-4, and IL-13, Peprotech) for 24 h, to induce M2 phenotypes. To affirm the anti-inflammatory potential of NiM-FA, we further investigated the ability of NiM-FA in reprogramming M1 macrophages. Two treatment conditions were tested. First, M1 polarized macrophages were treated with NiM-FA (25 µg/mL of Zol) for 24 h and 48 h and this condition was regarded as NiM-FA post-M1 activation. Second, RAW 264.7 macrophages were first treated with NiM-FA (25 µg/mL of Zol) for 24, washed then polarized to M1 as previously described using LPS + IFN γ and referred to as NiM-FA pre-M1 activation. Post polarization, the cells were blocked for FC receptor in TruStain FcX™ PLUS (anti-mouse CD16/32,Biolegend) for 10 min on ice, at 0.25 µg per 10^6^ cells in a volume of 100 µL, and without any washing, immunostained for 30 min on ice.

Thereafter, macrophage phenotype was assessed via confocal microscopy and flow cytometry using typical surface markers (PE-CD86 for M1 and FITC-CD206 for M2 , Biolegend). Unstimulated cells served as unpolarized controls (Mф macrophages). Additionally, changes in cell size associated with polarization were validated through forward scatter (FSC) and side scatter (SSC) analysis.

### Statistical analysis

Data analysis and figure drawing were completed using GraphPad Prism 9.5.1 (GraphPad Software, Inc. San Diego, CA, USA), and all quantitative data were expressed as mean ± SD from at least triplicate measurements. Differences between two comparative groups were analyzed using the student’s t-test, while significance among multiple groups was evaluated using one-way or two-way analysis of variance (ANOVA), followed by a recommended post-hoc multiple comparison test as detailed in the figure legend. Statistical significance was determined at a 95% confidence level with the following thresholds: *p* < 0.05 (*), *p* < 0.01 (**), *p* < 0.001 (***), and *p* < 0.0001 (****).

## Results

### Preparation and characterization of CaZol NPs and CaZol NiM

**Fig. 1:**
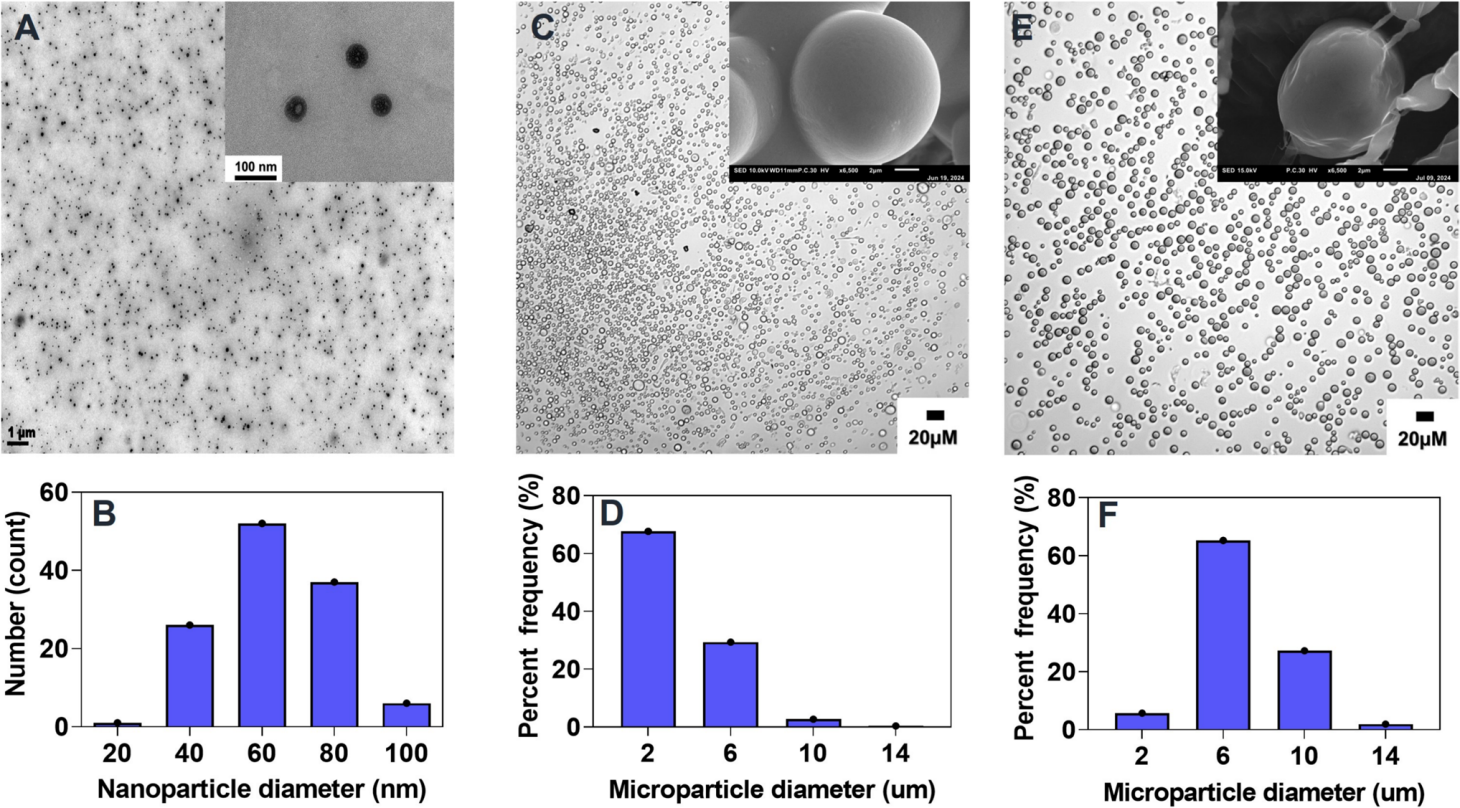
Particle size distributions of nanoparticles and microparticles. Micrographs and histograms of particle size distributions of **A&B)** CaZol NP, **C&D)** PEG-PLGA MP, and **E&F)** CaZol NiM

DOPA-coated CaZol NP was successfully synthesized by reverse microemulsification. As shown in Fig. 1A&B, the nanoparticles synthesized displayed uniform morphology and particle size with normal distributions. The number-average diameter (Dn) calculated based on TEM images was 63.5 ± 17.2 nm (Table 1) which was very close to the hydrodynamic diameter (Dh) determined by DLS. Particularly, CaZol NP showed a narrow particle distribution (PDI of 0.2) with a hydrodynamic particle size of ~ 82 nm, and a corresponding particle concentration of 1.39 × 10^13^ (Fig. S1A&B). The CaZol NP had a zeta potential of -10.0 ± 0.6 mV which indicates the presence of an anionic surface coating like DOPA (Fig S1C).

**Table 1:** Encapsulation efficiency, drug and nanoparticles loading capacity of formulations

Formulation	Encapsulation efficiency (EE%)	Drug loading (DL%)	Nanoparticles per microparticle
CaZol NP	45.1 ± 0.4	4.9 ± 0.1	-
CaZol NiM	55.8 ± 9.0	3.9 ± 0.1	32 ± 12

PEG-PLGA MP and CaZol NiM (PEG-PLGA MP loaded with CaZol NP; CaZol NiM) were successfully fabricated using the coaxial flow phase separation technique. As shown in Fig. 1, both displayed homogeneous particle sizes with normal distributions. PEG-PLGA MP showed an average size of 4.0 ± 1.4 um with a PDI of 0.35. Addition of CaZol NP to generate CaZol NiM led to a slight but significant increase in the average size of MPs to 6.9 ± 2.1 μm with a PDI of 0.30 (Suppl Table 1 and Fig. 1F and S2**)**. A similar increase in size of microparticles was also observed when Zol was directly loaded onto PEG-PLGA MP (PEG-PLGA Zol MP).

**Fig. 2:**
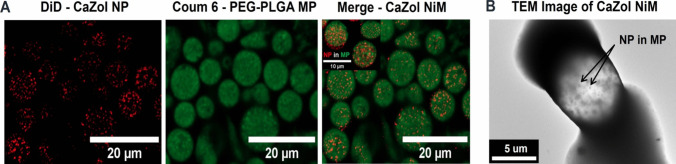
Microscopy characterization of CaZol NiM. **A)** Confocal images of CaZol NP (DiD labeled), PEG-PLGA MP (Coum-6 labeled) and merged image CaZol NiM showing CaZol NP encapsulated within MP. **B)** TEM image of CaZol NiM

The successful encapsulation of CaZol NP within microparticles was further verified utilizing a combination of direct imaging and indirect chemical composition analysis. Confocal imaging of the CaZol NiM revealed uniform distribution of CaZol NP within the microparticles, as seen in Fig. 2A. To mitigate concerns regarding dye leaching and potential intermixing, non-dye labeled particles were examined via TEM to confirm the presence of the CaZol NP within the microparticles (Fig. 2B).

**Fig. 3:**
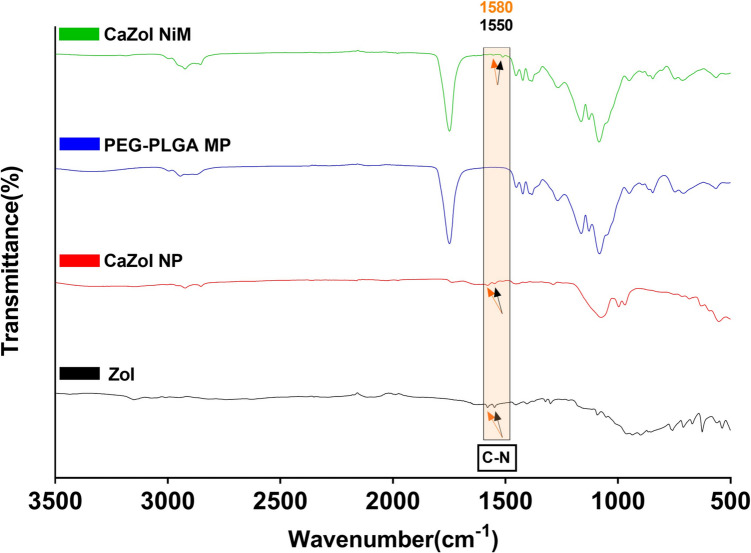
FTIR spectra of CaZol NiM, CaZol NP, PEG-PLGA MP, and free Zoledronic acid

Further analysis of chemical composition through FTIR (Fig. 3), of free Zol and Zol loaded particles (CaZol NPs and CaZol NiM) showed a characteristic C-N stretching band at 1550 cm^−1^ and 1580 cm^−1^ and a broad C = C stretching band at 1452 cm^−1^ due to the vibration in the imidazole ring in Zol, while these bands were absent in the empty PEG-PLGA MP (blank). Moreover, the presence of the phosphate group in Zol and CaZol NP and CaZol-NIM was confirmed by 1150 cm^−1^ and 1070 cm^−1^ bands which correspond to P = O and a P-O stretching respectively [7, 42]. The strong band at 1748 cm^−1^ in the PEG-PLGA MP, CaZol NiM corresponds to the C = O stretch due to presence of PLGA, while the characteristics peak at 1083 cm^−1^ showing the C–O–C stretch confirms the presence of PEG. Additionally, phosphorus was utilized as a marker to quantitatively assess the presence of Zol and CaZol NP within MPs. This was achieved through EDAX analysis, wherein the amount of phosphorus (P) relative to carbon (C) and oxygen (O) was determined for CaZol NP, CaZol NiM, and PEG-PLGA MPs (control). The results indicated phosphorus percentages of 17.03 mol %, 0.86 mol %, and 0 mol % for CaZol NP, CaZol NiM, and PEG-PLGA MPs, respectively (Fig. S3). Similarly, the high presence of chlorine and calcium representative of nanoparticles presence was observed in CaZol NP, while minimal amounts were detected in the NiM formulation indicating shielding due to successful encapsulation. However, these elements were not detected in the blank PEG-PLGA MP control sample (Fig. S3). Collectively our data indicates successful encapsulation of CaZol NP in MP and fabrication of CaZol NiM.

### Encapsulation efficiency, drug and nanoparticles loading capacity, and release kinetics of CaZol NP and NiM

The average number of nanoparticles loaded into a given microparticle was determined as 32 ± 12 (Table 1). CaZol NiM showed a relatively higher encapsulation efficiency compared to CaZol NP (Table 1). Notably loading Zol as CaZol NP doubled the encapsulation efficiency compared to free Zol loading in PEG-PLGA MP (Suppl. Table 1). All particle formulations showed similar percentage of drug loading (Table 1 and Suppl. Table 1). Further we evaluated drug release kinetics from CaZol NP and CaZol NiM only, owing to their similar encapsulation and drug loading efficiency and pH responsiveness of the calcium complexed Zol NP.

**Fig. 4:**
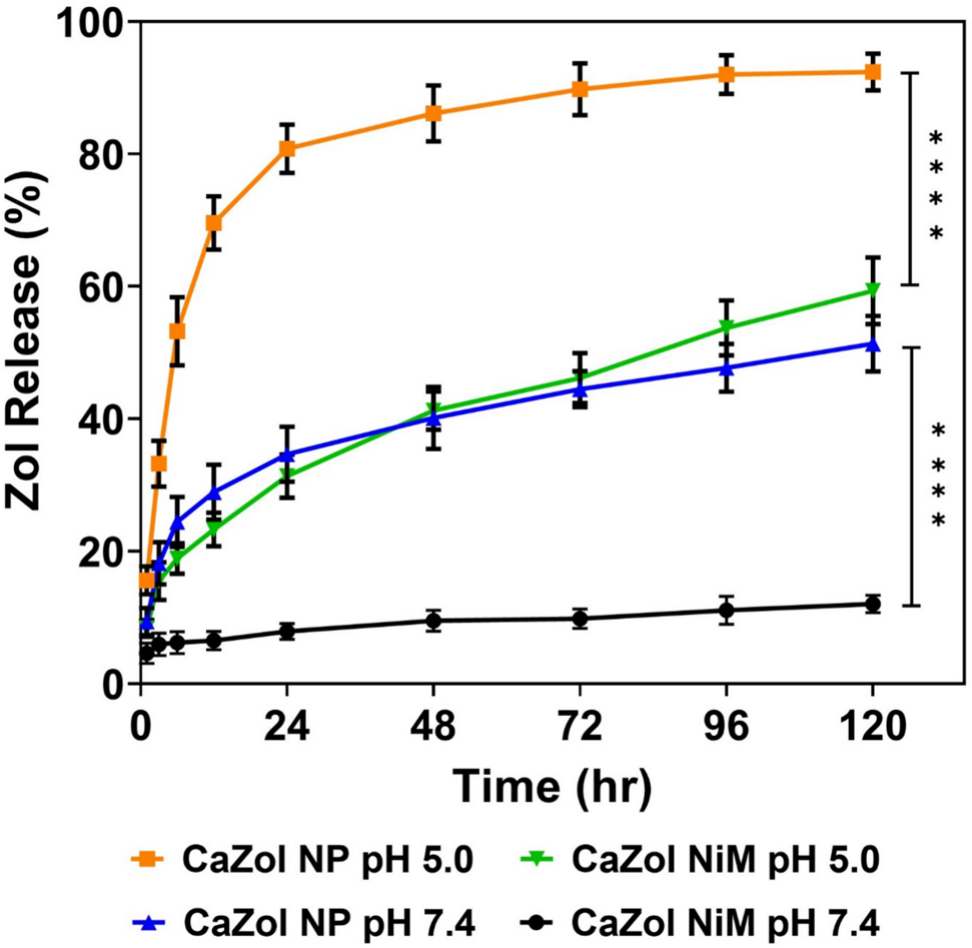
Kinetics of zoledronic acid release from CaZol NP and CaZol NiM in PBS buffer at pH 7.4 and pH 5. N = 3 for all experiments. Two-way analysis of variance (ANOVA), followed by Tukey’s post-hoc multiple comparison test, was used to assess the significance of Zol release from CaZol NP and CaZol NiM independently at 24 h and 120 h under the same pH conditions (either 7.4 or 5.0). Results showed significant difference indicated by *p* < 0.0001, (****)

Both CaZol NP and CaZol NiM demonstrated sustained Zol release over a 5-day period at pH 7.4, but rapid release when incubated in a buffer of pH 5.0 (Fig. 4). Notably, CaZol NiM exhibited superior control over burst release in both acidic and neutral conditions compared to CaZol NP. Specifically, at pH 7.4, CaZol NiM released approximately 8% of Zol within 24 h, compared to 35% of Zol released from CaZol NP (p < 0.0001). Similar release behavior was observed at same pH condition after 120 h, with CaZol NiM releasing approximately 12% of Zol while CaZol NP released approximately 51% of Zol (p < 0.0001). Also, at pH 5.0 after 24 h, CaZol NiM released approximately 31% of Zol, whereas CaZol NP released about 81% of Zol (p < 0.0001). Similarly, by 120 h at same pH of 5.0, CaZol NiM released approximately 59% of Zol, whereas CaZol NP released about 92% of Zol (p < 0.0001). These findings underscore the pH-responsive nature of these particles, indicating that CaZol NiM can remain stable at physiological pH found in tissue fluids while exhibiting rapid release in acidic compartments such as lysosomes. This attribute is advantageous as it minimizes non-specific drug release at physiological pH and facilitates lysosomal escape following cellular uptake and intracellular drug release [43].

Further, in a long-term release study assessed by Inductively Coupled Plasma Optical Emission Spectroscopy (ICP-OES), CaZol NiM showed sustained Zol release over 12 days, with only 40% of the drug released at pH 5.0. In contrast, CaZol NP released 100% of the drug by day 7. At pH 7.4, both particle types displayed a lower percentage of drug release, with CaZol NiM releasing less than 10% of Zol over the 12-day period (Fig. S4A). The release of the CaZol NP from the CaZol NiM was also confirmed through imaging where both CaZol NiM and CaZol NP were captured via TEM (Fig. S4B). Notably, minimal disintegration of the CaZol NP was observed when CaZol NP was incubated in neutral medium (chloroform) and PBS with physiological pH of 7.4 (Fig. S5). However, in a pH 5.0 PBS buffer, CaZol NP was observed to disintegrate leading to a decline in NP size (Fig. S5). The pH dependent prolonged release and high stability at physiological pH also indicates strong chelation of Zol with calcium.

### In vitro cellular uptake of Zol-loaded particles

**Fig. 5:**
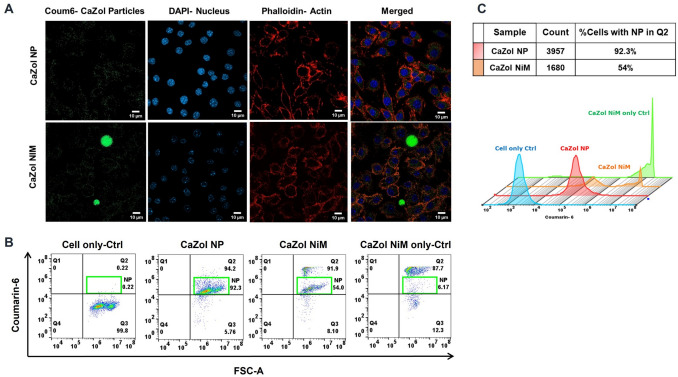
Cellular uptake of CaZol NP and Ca Zol NiM by RAW 264.7 macrophages. **A)** Cellular uptake of CaZol NP and CaZol NiM as observed via confocal microscopy. Green Coum-6-stained nanoparticles, blue: nucleus (DAPI), red: Phalloidin-actin; **B&C)** Quantitative determination of the particle uptake by macrophages by flow cytometry and characterization of percentage of CaZol NP and CaZol NP released from NiM upon cellular internalization

Both CaZol NP and the CaZol NP released from CaZol NiM were effectively taken up by the activated RAW 264.7 macrophages as shown in Fig. 5. Although CaZol NiM was taken up by macrophages less rapidly than CaZol NP, direct uptake of the microparticles was observed. Additionally, a controlled release of CaZol NP from the NiM was evident, resulting in qualitatively lower cellular uptake of CaZol NP (Fig. 5A). This observation was quantitatively supported by flow cytometry, which showed a higher uptake of coumarin 6-labeled CaZol NP compared to the NP released from CaZol NiM (Fig. 5B, C). Notably, two distinct peaks corresponding to the uptake of both released CaZol NP and microparticles were visible, aligning with their positions relative to the reference samples of CaZol NP and NiM-only controls. Also of evidence is an ‘NP’ gated population highlighted in green representing the percentage of cells that had distinctly taken up CaZol NPs. As shown in (Fig. 5B, C), while nearly all the cells in region Q2 were positive for coumarin-6 due to unanimous uptake of NP, treating the cells with CaZol NiM resulted in a general decrease in total count to 1680, and a reduction in the percentage of cells positive in the NP gate to 54%. These results are consistent with the release study findings, confirming that the NiM formulation provides sustained and controlled release of CaZol NP, and subsequently Zol.

### Targeted delivery via ligand mediated uptake

We further evaluated the capability of the CaZol NiM system to be used for targeted delivery to macrophages, which are notable for overexpressing folate receptor (FR) [44, 45]. Due to potential overlap of spectral peaks of functional group distinct to folic acid (FA) and Zol, blank microparticles were first synthesized and characterized for successful ligand incorporation. The presence of FA on PEG-PLGA microparticles was confirmed by FTIR analysis, with characteristic peaks observed at 1574 cm⁻^1^ (N–H bending), 1625 cm⁻ [1] (C = C bending), and 3423 cm⁻ [1] (N–H amine stretching), indicating the successful incorporation of FA as a ligand (Fig. S6).

**Fig. 6:**
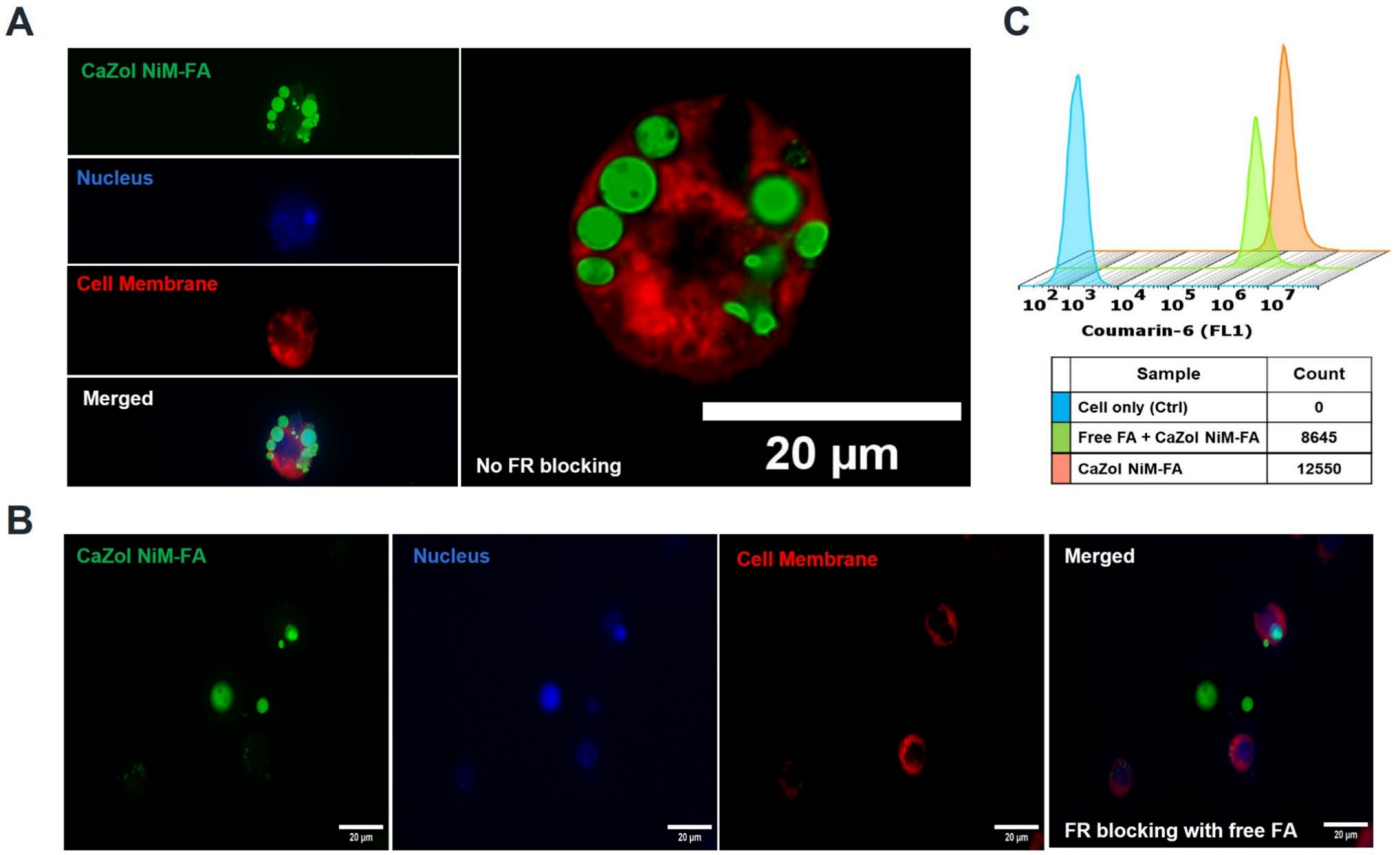
Targeted uptake of CaZol NiM-FA by macrophages. Confocal imaging of the NiM uptake by macrophages **A)** no pretreatment by FA; **B)** pre-treatment of cells with 1 mM FA to block FR receptor. **C)** Flow cytometry histograms quantifying the uptake of NiM in macrophages pre-treated and not treated with FA

Further loading of CaZol NP into FA-PEG-PLGA microparticles resulted in the synthesis of CaZol NiM-FA, which upon incubation with macrophages, showed enhanced uptake (Fig. 6A). Pretreatment of the cells with free FA resulted in reduction of cell uptake of NiM as observed by confocal imaging and flow cytometry, further confirming FA mediated targeted uptake of NiM (Fig. 6B, C).

### Intracellular trafficking and lysosomal localization of particles

**Fig. 7:**
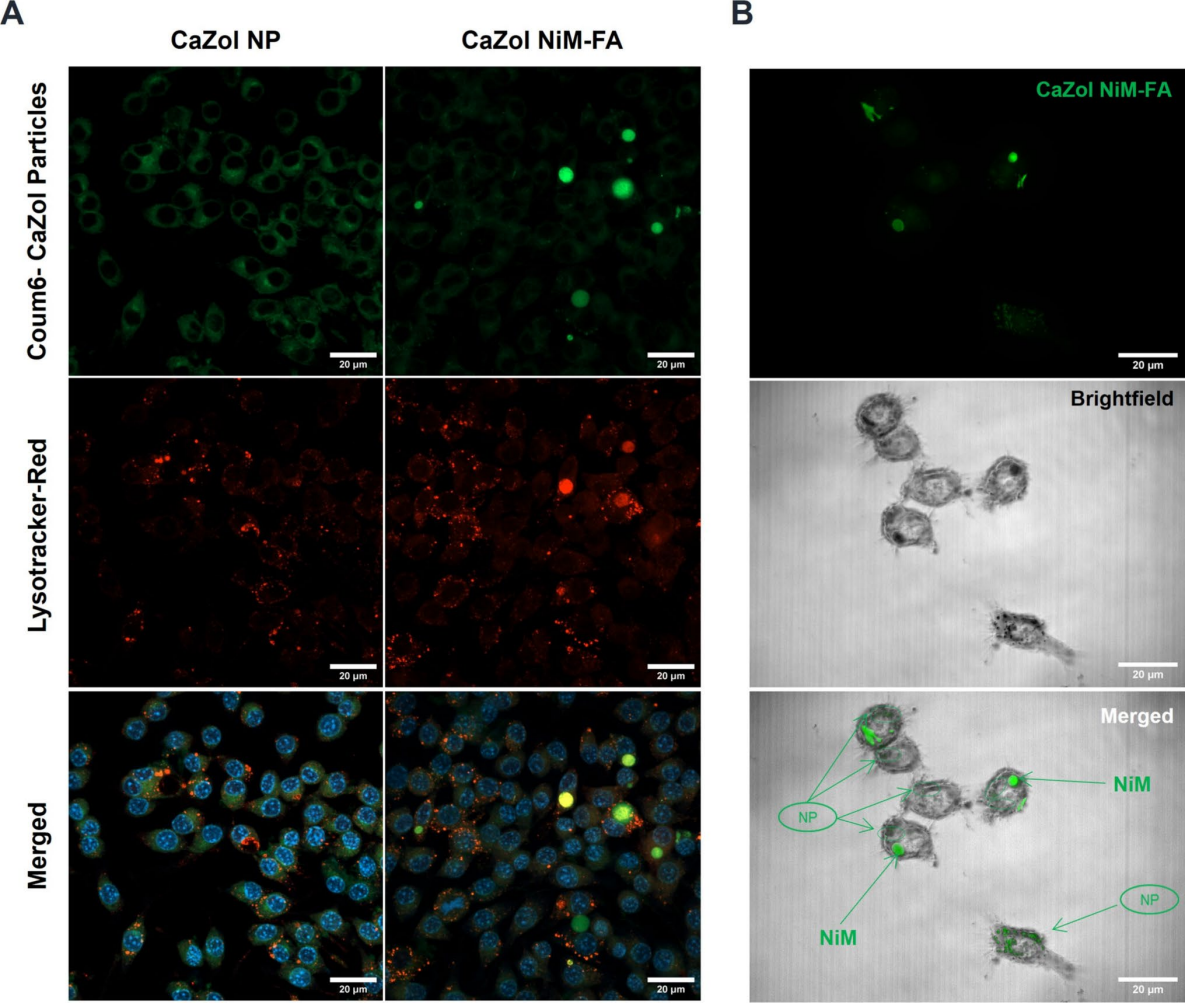
Intracellular localization of the CaZol NiM-FA and CaZol NP**. A)** Confocal images of RAW macrophages when incubated with either CaZol NP or CaZol NiM-FA and immunostained with lysotracker dye. The merged panel shows orange where coum6- stained particles colocalize with red stained lysosomal/acidic compartments with cell. **B)** Intracellular trafficking of CaZol NiM-FA showing release of NP from NiM after uptake

Both CaZol NiM and free CaZol NP, colocalized with the lysosomal compartment of the cell, as monitored by a lysosomal tracker dye **(**Fig. 7A**, **Fig. S7**)**. Further investigation of the ability of NiM to intracellularly control the release of CaZol NP was confirmed by the presence of both NP and NiM in the cells as shown by green arrows in Fig. 7B**,** indicating that NiM upon uptake can favorably act as an intracellular depot to regulate the mobility and release of CaZol NP. Collectively, these results indicate that the NiM system not only is beneficial for minimizing the rapid release of Zol at physiological pH but also underscores the potential of the system to capitalize on its pH sensitivity to drive the release of CaZol NP in a controlled manner upon localization with the acidic compartment of the lysosome.

### Effects of Zol on macrophage viability in vitro

**Fig. 8:**
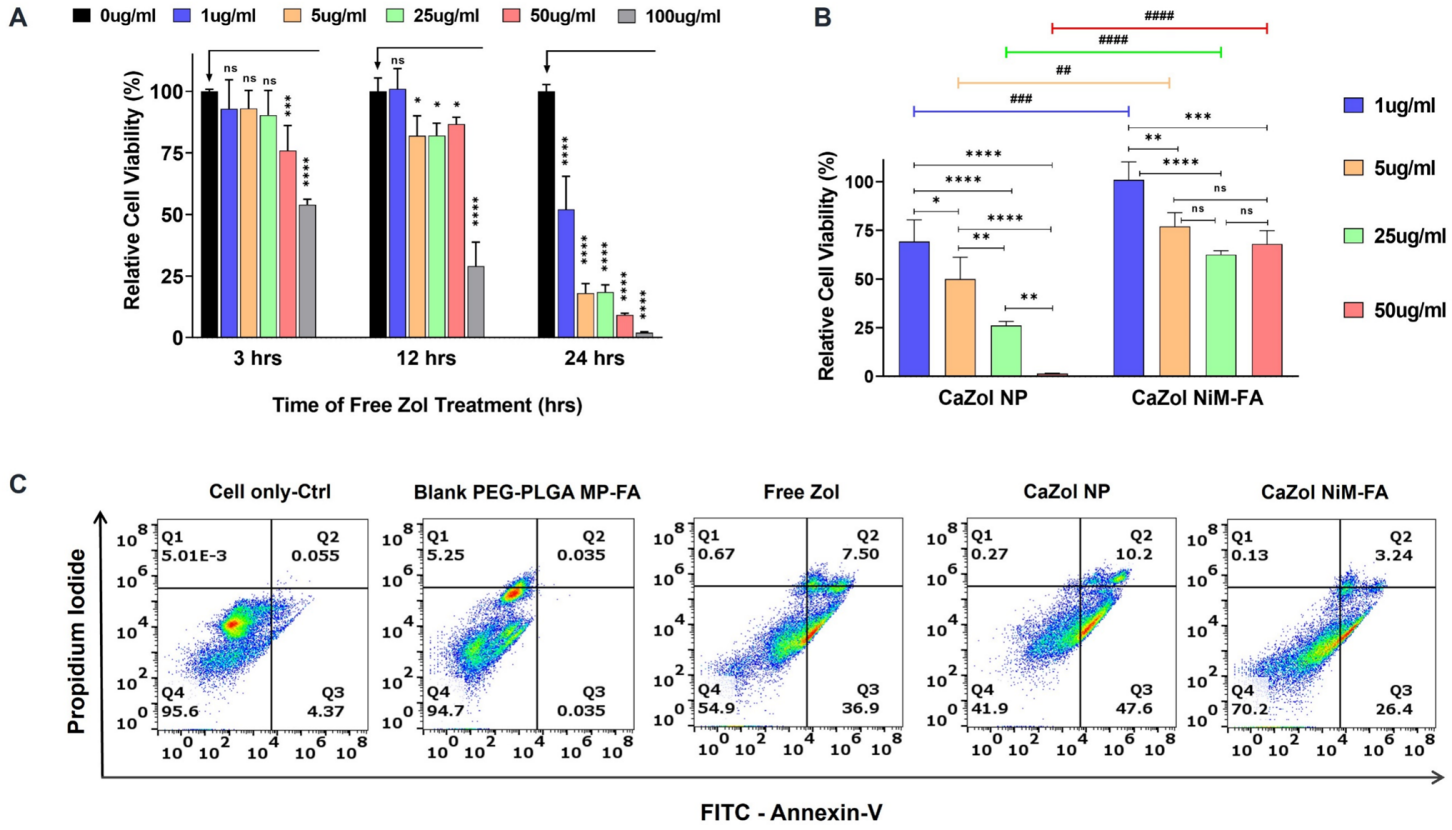
Effects of Zol and Zol loaded particles on cell viability. **A)** Cell viability assessment by MTT assay after treatment with different doses of Zol for various treatment periods. **B)** Cell viability after 24 h treatment with CaZol NP and CaZol NiM-FA having equivalent Zol concentration ranging from 1- 50 μg/mL. **C)** Flow cytometry quantification of apoptosis in RAW 264.7 macrophages upon 24 h treatment with CaZol NP and CaZol NiM-FA having a Zol equivalent concentration of 25 μg/mL. Two-way ANOVA followed by a recommended post-hoc test was used for comparing significant differences in multiple groups. In Fig. 8A, the symbol (*) indicates significance determined by Dunnett's multiple comparison test, comparing different concentrations of Zol within the same treatment time group relative to the 0 μg/mL blank control. In Fig. 8B, the symbol (*) indicates significance determined by Tukey's multiple comparison test, comparing among different concentrations of Zol within the same Zol-particle formulation. Similarly, the symbol (#) represents significance determined by the Bonferroni test, comparing the same Zol concentrations across different Zol-particle formulations. Statistical significance was determined at a 95% confidence level, with the symbolic index (* or #) defined base on the *p* values thresholds indicated in the statistical analysis section in methods

To determine the optimal dose and treatment duration of Zol that induces the highest toxicity in macrophage cells, various concentrations of free Zol were tested over different treatment periods. MTT assay results indicated that Zol at 100 μg/mL consistently exhibited the greatest toxicity, with the lowest cell viability after 24 h of treatment across all tested concentrations (Fig. 8A). Consistent with these findings, 24-h treatments using CaZol NP and CaZol NiM-FA confirmed a dose-dependent toxicity response. Interestingly, CaZol NiM-FA had a minimal impact on cell viability across different doses, likely due to its enhanced ability to release Zol in a sustained manner over time (Fig. 8B). This sustained release and targeting capacity of the NiM platform was further validated by our assessment of cytotoxicity between non-targeted CaZol NiM and targeted CaZol NiM-FA, where CaZol NiM exhibited a higher viability of ~ 82% compared to ~ 61% shown by CaZol NiM-FA. (Fig. S8**).** This may be due to higher receptor mediated uptake of the CaZol NiM-FA via cells as compared to non-targeted NiM, leading to higher intracellular drug release and cytotoxicity.

MTT results were further validated by flow cytometry analysis of apoptotic cells using Annexin V/PI staining (Fig. 8C). After 24 h, approximately 70% of cells remained viable following treatment with CaZol NiM-FA, compared to 42% and 55% viability for cells treated with CaZol NP and free Zol, respectively. As a control, treatment with blank PEG-PLGA MP-FA, which lacks Zol, showed a high cell viability of approximately 95%, indicating biocompatibility of the PEG-PLGA MP. For further studies evaluating the anti-inflammatory effect of NiM we excluded free Zol and CaZol NP as controls owing to high cytotoxicity.

### Effect of CaZol NiM on NF-κB activation and reactive oxygen species generation

**Fig. 9:**
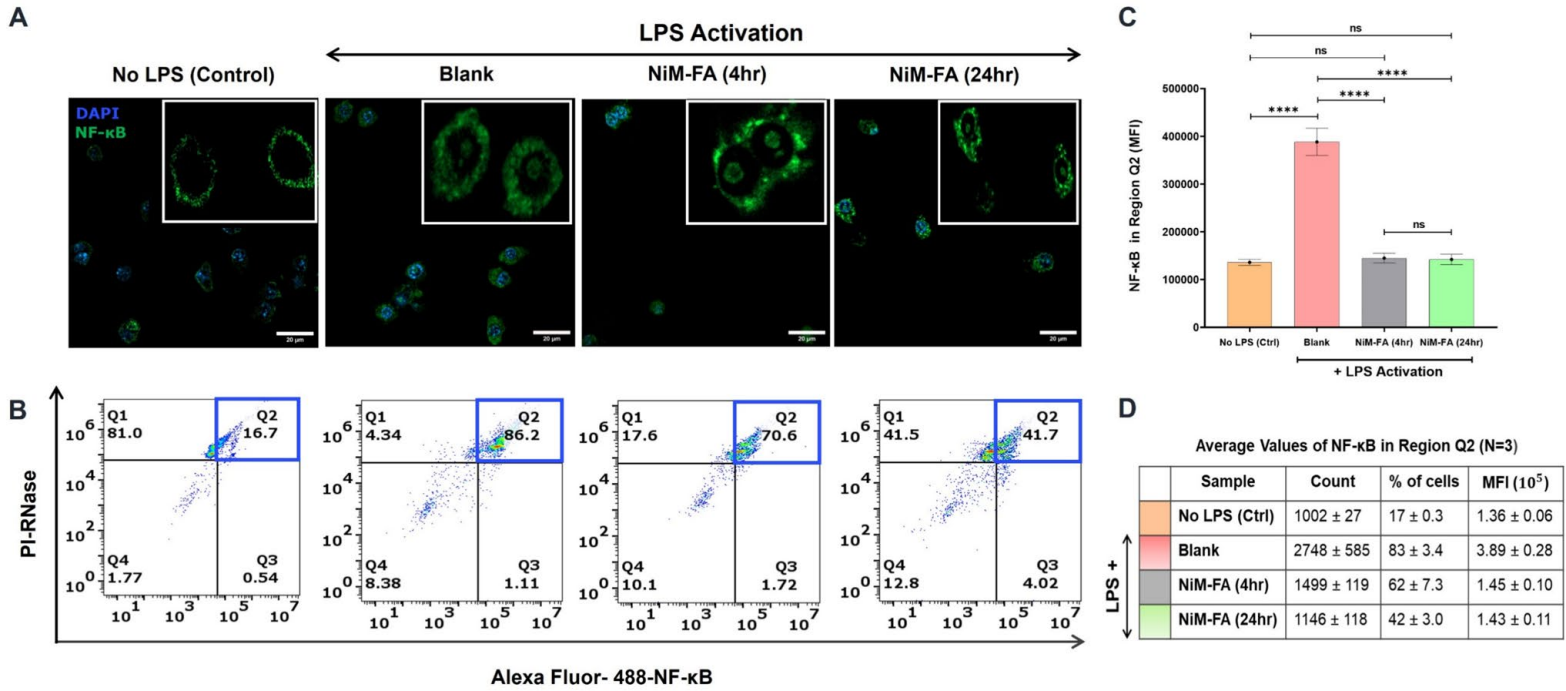
Effects of CaZol NiM-FA on NF-κB activity and inflammation. **A)** Confocal images of immunostained cells depicting intracellular location of NF-κB p65 in activated macrophages treated with CaZol NiM-FA for 4 and 24 h. **B)** Flow cytometry cytogram of nuclear colocalization of PI/RNase and Alexa Fluor 488-NF-κB in activated macrophages treated with CaZol NiM-FA for 4 and 24 h. **C)** Quantification of nuclear translocation and activation of NF-κB by comparing the MFI values of cells/ Q2 populations, double positive for PI/RNase and Alexa Fluor 488-NF-κB amongst different groups. **D) **Average values for count, % positive cells, and MFI of NF-κB in region Q2 across all sample condition. Two-way ANOVA was used for comparing multiple groups in **C**. Asterisks “*” represents significance computed by Tukey multiple tests to compare between different timepoints treated with CaZol NiM-FA formulation and LPS only treatment. N = 3 for experiments. *p* < 0.05 (*), *p* < 0.01 (**), *p* < 0.001 (***), and *p* < 0.0001 (****).

We examined the effect of CaZol NiM on NF-κB activation, a critical regulator of inflammation. Compared to control cells, LPS-activated RAW 264.7 macrophages showed the colocalization of the NF-κB p65 subunit with the nucleus, indicating nuclear translocation and activation of NF-κB (Fig. 9A). However, treatment of activated macrophages with CaZol NiM-FA reduced the nuclear-to-cytosolic ratio of p65, suggesting a decrease in NF-κB activation.

Flow cytometry analysis confirmed this finding, showing a reduction in mean fluorescence intensity (MFI) for phosphorylated NF-κB p65 (indicative of activated nuclear NF-κB) after 4 h of treatment (Fig. 9 B&C; Fig. S9: Gating strategy). The inhibitory effect of CaZol NiM was sustained at 24 h, with MFI levels remaining similar between the 4-h and 24-h and were similar to baseline levels as in non-activated control cells. Furthermore, the percentage of cells with activated NF-κB decreased from 62% at 4 h to 42% at 24 h, highlighting the anti-inflammatory potential of CaZol NiM (Fig. 9B–D).

Notably, CaZol NiM-FA emerged as the most effective formulation compared to free Zol and CaZol NP in suppressing NF-κB activation, achieving MFI levels comparable to non-activated control cells (Fig. S10).

As a control, we validated the ability of Zol and Zol-loaded particles to activate NF-κB without LPS stimulation. Minimal to no NF-κB activation was observed in RAW macrophages treated with CaZol NiM-FA or CaZol-NP (loaded with 25 μg/mL Zol) after 24 h, while the free Zol formulation exhibited some degree of nuclear colocalization (Fig. S11). This further underscores the anti-inflammatory efficacy of CaZol NiM-FA in mitigating NF-κB activation.

**Fig. 10:**
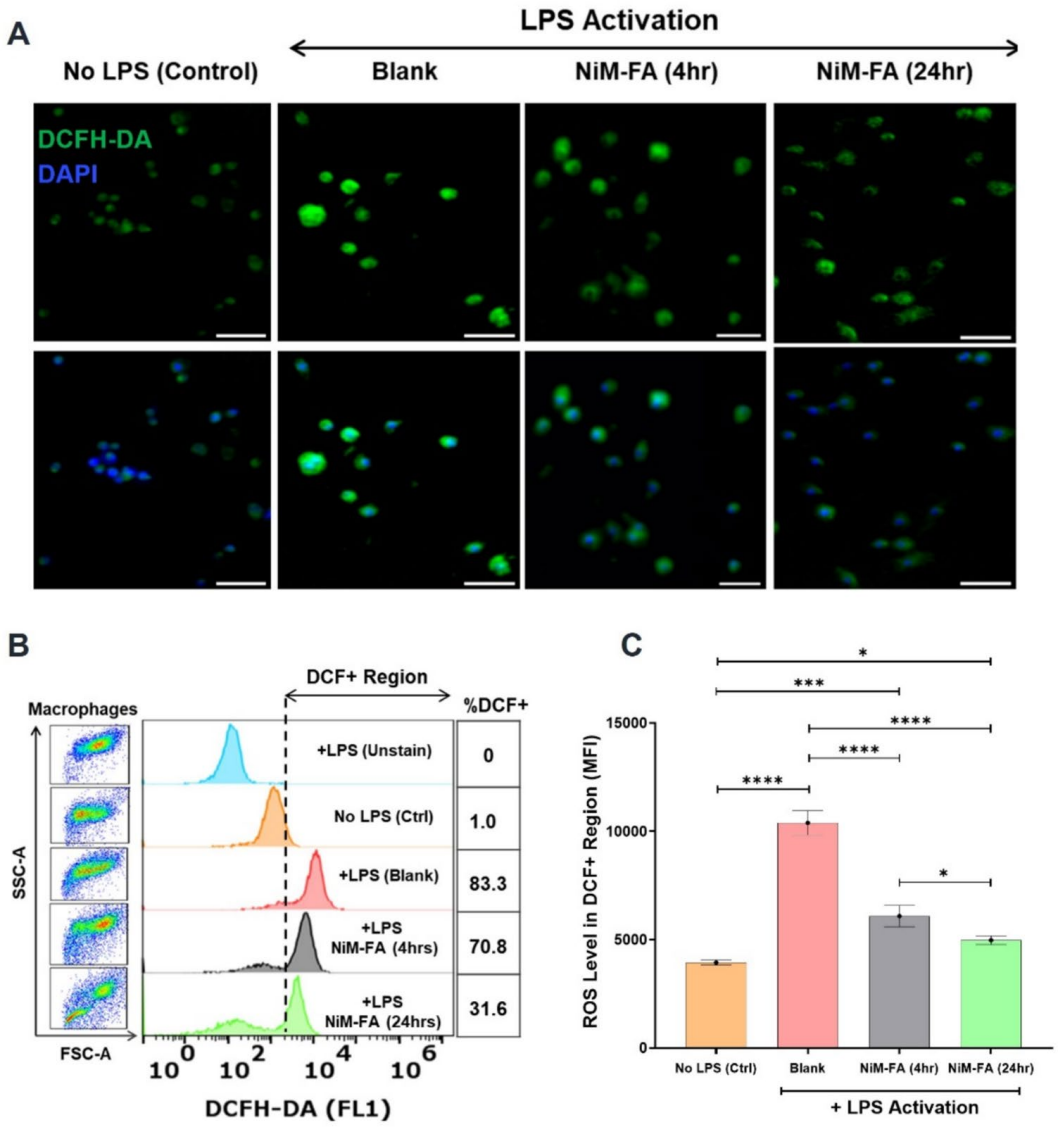
Effects of CaZol-NiM-FA on inhibiting ROS. **A)** ROS levels in cells as indicated by the generation of fluorescence signal after incubation with DCFH-DA dye in activated macrophages treated with CaZol NiM-FA for 4 and 24 h. **B)** Flow cytometry cytogram quantifying the level of fluorescence as measure of ROS levels in activated macrophages. **C)** Bar plot indicating the relative level of ROS in activated macrophages by comparing the MFI values of DCFH-DA dye amongst different groups. Two-way ANOVA was used for comparing multiple groups in **C**. Asterisks “*” represents significance computed by Tukey multiple tests to compare between different timepoints treated with CaZol NiM-FA formulation and LPS only treatment. N = 3 for experiments. *p* < 0.05(*), *p* < 0.01 (**), *p* < 0.001 (***), and *p* < 0.0001 (****).

Complementing the NF-κB inhibitory activity of CaZol NiM we also noticed a concomitant attenuation of ROS generation in activated macrophages. Following treatment with CaZol NiM, we observed a reduction in the intensity of ROS generation per cell (measured by MFI) in activated macrophages. This decrease became even more pronounced after 24 h of continuous treatment (Fig. 10A & C). Additionally, CaZol NiM treatment significantly reduced the percentage of ROS-positive cells, dropping from 73% after 4 h to just 31.6% after 24 h of treatment (Fig. 10B).

### Effect of CaZol NiM on macrophage repolarization

Encouraged by the demonstrated anti-inflammatory effect of CaZol NiM-FA, we further probed into its potential for re-educating macrophage polarization states. We first polarized RAW 264.7 macrophages to M1 and M2 states and confirmed their distinct states by observation of morphological differences and expression of surface markers, CD86 and CD206. Notably, while Mф macrophages showed minimal to no expression of either of the conventional phenotypic surface markers, the M1 and M2 macrophages showed preferential expression of CD86 and CD206, respectively (Fig. S12) [46]. Upon polarization, M1 macrophages adopted a flattened and spindle shape, while Mф and M2 macrophages appeared rounded and circular (Fig. S13), as reported previously [46]. Given that a key measure of macrophage activation is the change in cell size, we also demonstrated from FSC vs. SSC plot in flow cytometry that M1 activated macrophages had significantly larger size than Mф, and M2 (Fig. S13).

**Fig. 11:**
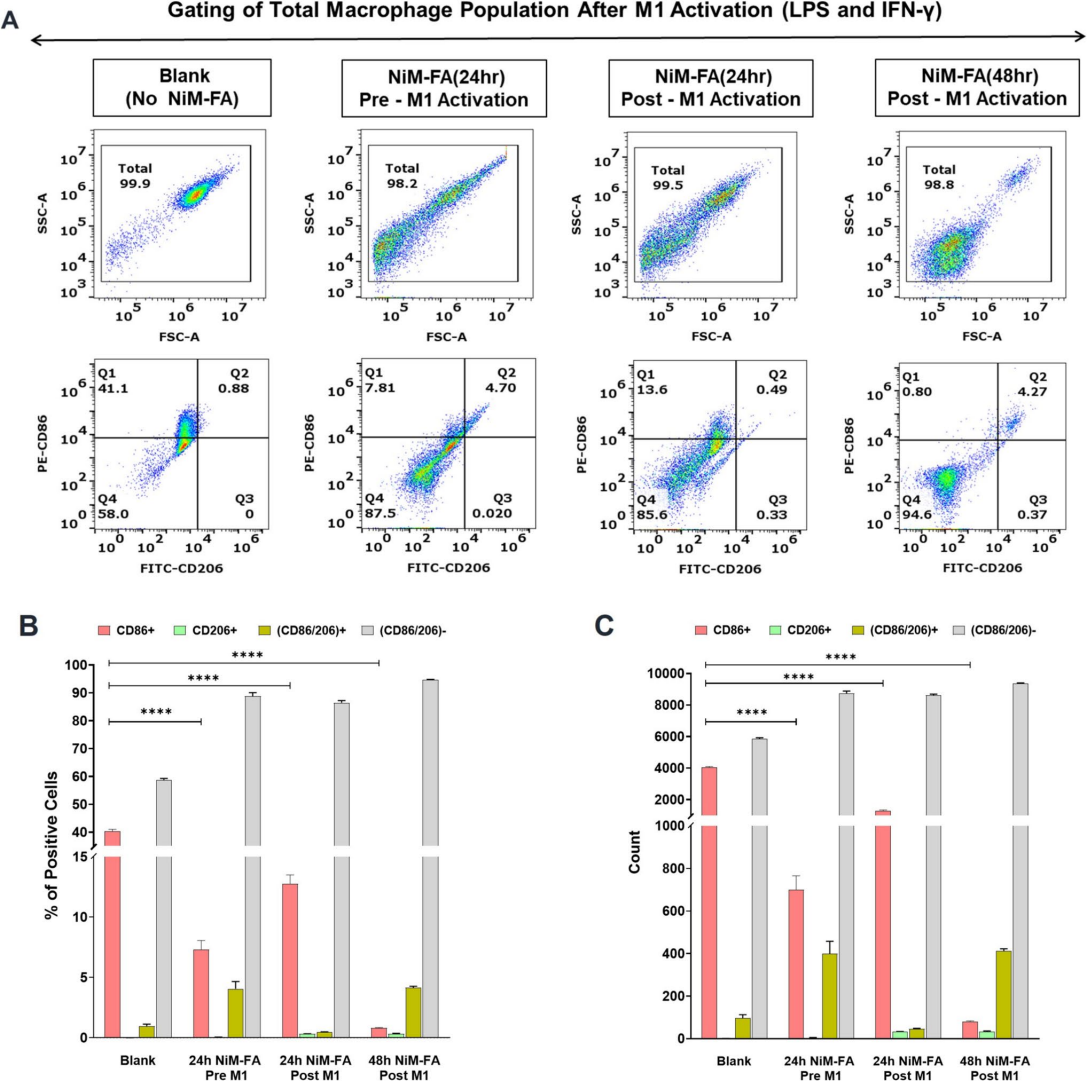
Effects of CaZol-NiM-FA on M1 Macrophage Repolarization. **A, B, and C)** Flow cytometry plot and bar graph representations showing expression in terms of % positive cells (**B**) and count (**C**) of CD 86 (M1 marker) and CD 206 (M2 marker) by M1 polarized cells (LPS + IFN-γ) with or without CaZol-NiM-FA treatment for 24 h or 48 h (Pre-M1 and Post M1 activation). Two-way ANOVA was used for statistical comparison across multiple groups in Fig 11**B, and C**. Asterisks “*” represents significance computed by Tukey multiple tests to compare between CD86^+^ values of blank and CD86^+^ values of treatment groups. N = 3 for experiments. *p* < 0.0001(****)

After establishing the expression markers of M1 and M2 phenotypes in RAW macrophages, we next evaluated the effect of CaZol NiM-FA on the repolarization of M1 macrophages. In activated macrophages, a distinct population of CD86⁺ M1 cells was observed, characterized by a high percentage of positive cells and an elevated count index (Fig. 11A–C). However, pre-treatment of these activated macrophages with CaZol NiM-FA for 24 h resulted in a significant ~ sixfold reduction in CD86⁺ M1 macrophages (Fig. 11A, B). Notably, we also observed a small but statistically significant increase of approximately 5% in CD86⁺/CD206⁺ cells, suggesting the emergence of an M2-like phenotype.

A similar shift in macrophage polarization, marked by a decrease in CD86⁺ cells, was observed when macrophages were first activated and then treated with CaZol NiM-FA for either 24 or 48 h. In this post-M1 activation treatment group, we noted a minor increase in CD206^+^ expression—0.33% and 0.37% for 24 h and 48 h treatments, respectively—compared to minimal expression in the blank control and pre-M1 activation treatment groups (0% and 0.02%, respectively), as shown in region Q3 of the dot plot (Fig. 11A). However, at prolonged exposure, a ~ 5% rise in CD86⁺/CD206⁺ cells were observed after 48 h of NiM-FA treatment, indicating a transition toward an M2-like phenotype.

Interestingly, a consistent trend was observed across both pre- and post-M1 activation treatment groups: a significant increase in macrophages that were double-negative for CD86 and CD206. These cells likely represent a steady-state or resting macrophage (Mф) phenotype, as evidenced by their low granularity in FSC vs. SSC cytograms, closely resembling that of control macrophages (Fig. 11 and S.13B). Collectively, these results demonstrate the potential of CaZol NiM-FA in repolarizing M1 macrophages toward a non-inflammatory state, thereby increasing their potential to acquire M2 phenotype. A detailed panel describing the gating strategy and experimental controls of Fig. 11 is shown in the supplementary (Fig. S14).

## Discussion

In this work, we complexed Zol to calcium to create pH-responsive nanoparticles (CaZol NP) and showed the potential for sustained and targeted delivery of Zol by loading these nanoparticles into PEG-PLGA microparticles fabricated using a customized coaxial flow phase separation method [40] to establish the CaZol NiM. Our method shows that loading Zol in the form of CaZol NPs into microparticles, rather than as free Zol, can double the encapsulation efficiency. This improved efficiency is likely due to the stability of CaZol NPs in the microemulsion, which ensures their effective encapsulation in the polymer. We selected calcium for nanoparticle synthesis due to the well-established high affinity of Zol for calcium ions [47], leading to stable nanocomplex formation between Zol and calcium. This choice was further motivated by physiological relevance: pre-complexing zoledronic acid (Zol) with calcium aims to reduce its binding to endogenous bone calcium, limit off-target skeletal accumulation, and improve delivery to non-skeletal, disease-relevant tissues. Further, the hydrophilic nature of free Zol may favor it to interact with the aqueous phase of the emulsion, during microparticle fabrication, thus decreasing its encapsulation in the polymer. This suggests that our approach can effectively overcome the limitations of conventional microencapsulation methods of encapsulating Zol into particulate systems [48]. Compared to earlier reported methods relying on modifying the polymer or lipid composition to achieve high encapsulation efficiency [8, 23, 49], our method provides a modular approach for tuning loading capacity and encapsulation efficiency often enabling us to achieve higher and comparable loading efficiency of Zol even with lesser amount of Zol used in the formulation. Mostly because Zol can be pre-complexed with calcium in desired amounts to form the nanoparticles and then encapsulated at indicated dose levels within the microparticles, this approach minimizes drug loss while offering additional control on the amount of Zol encapsulated. This is particularly advantageous for scaling up production of CaZol NiM given that, our previous work [50] on microparticles synthesis confirms that fabrication parameters including polymer concentration, surfactant concentration, stir rate of the microemulsion, the volume of both organic and aqueous phase, and others can be carefully varied to control microparticles characteristics with batch-to-batch consistency and high reproducibility allowing for encapsulation of sufficient payloads without compromising the intended size of the microparticle system.

To gain further insight into the mechanisms underlying the release of Zol, and the extent to which our system can promote control and sustain release, we investigated the release behavior of Zol at neutral and acidic pH (7.4 and 5.0). Notably, our results show that both CaZol NP and CaZol NiM formulations exhibit a pH-responsive release, with the acidic medium aiding higher release of Zol as compared to the neutral pH condition. Our finding corroborates earlier reports and can be attributed to the weakened chemical coordination between calcium cations and the phosphate anions of Zol, owing to the protonation induced by the acidic environment [23, 51]. To substantiate this observation, our TEM results revealed that after 7 days, CaZol NP in an acidic medium (pH 5.0) showed noticeable particle degradation, significant morphological changes, and a reduction in size, compared to neutral medium (pH 7.4) and chloroform (Fig. S5). Furthermore, it is worth mentioning that as compared to CaZol NP, CaZol NiM effectively minimized the burst release of Zol at both pH conditions, consequently prolonging the release of Zol over longer period (Fig. 4 and Fig. S4) and reducing cytotoxicity caused by burst release (~ 35% in 24 h) from CaZol NP (Fig. 4 & 8). This could be due to the additional barrier provided by the microparticle platform as the released Zol from CaZol NP, will have to diffuse out of the polymer matrix before getting into the release medium. Therefore, these findings coupled with the ability of CaZol NiM-FA to colocalize with lysosomes, an acidic compartment as shown (Fig. 7), indicate that CaZol NiM-FA might release Zol at therapeutic concentrations only upon cellular uptake and reduce cytotoxicity. This underscores the potential application of CaZol NiM for spatially targeted delivery, where just a minimal amount of Zol is expected to be released from particles during their long-term use at physiological pH, thus reducing the risk of side effects of Zol on surrounding tissues. Our NiM system further corroborates advantages of multifunctional systems incorporating nanoparticle within microparticles [37–39] and allowing highly controlled drug release even in acidic conditions.

To enhance the targeting ability of CaZol NiM we choose folic acid as targeting ligand, which demonstrated excellent targeting ability and high uptake in activated macrophages (Fig. 6). Further, the uptake of CaZol-NiM was reduced significantly when free folic acid was added to the medium to block the folate receptors. Folic acid was chosen as ligand because of the high affinity of the folate for the folate receptor Kd < 10^–9^ [52–55]. Folate receptors (FR) are known to be overexpressed by activated macrophages in tissues affected by cancer and arthritis [44, 45]. Moreover, exclusive expression of the folate receptor-2 (FR-2) in the activated macrophages present at sites of chronic inflammation, including in rheumatoid arthritis (RA), osteoarthritis (OA), atherosclerosis, and inflammatory bowel disease (IBD) makes it an excellent candidate for targeted delivery to macrophages. By contrast, FR-1 is typically expressed on epithelial cells but has also been implicated in certain disease-associated macrophage subtypes and is overexpressed in many cancers, making it a useful dual-target for inflammatory and cancer-associated macrophages. Thus, including folate as the ligand on the CaZol NiM further enhances the targeting ability of our system by synergizing the macrophage targeting affinity of Zol, a bisphosphonate drug and folic acid and potentially reducing off target distribution to other cell types.

A further potential benefit of the CaZol NiM over free Zol or CaZol NP is the highly reduced cytotoxicity of Zol. Our results indicate significant cytotoxicity for Zol even at clinically relevant dose of 1 μg/mL (~ 3 μM) after 24 h of treatment (Fig. 8A). These results corroborate earlier findings that Zol exhibits significant cytotoxicity at higher concentrations and prolonged exposure times (Fig. 8A), as reported in previous studies [6, 23, 56, 57]. Contrastingly, delivery of Zol via CaZol NiM reduced toxicity of Zol even at elevated doses equivalent to 25 µg/mL (~ 90 μM) (Fig. 8B) after 24 h treatment. These results indicate that CaZol NiM can efficiently sequester Zol and serve as a depot for sustained delivery of Zol at a highly controlled rate, which can extend the use of Zol for treating macrophage mediated chronic inflammatory diseases affecting skeletal and non-skeletal tissue and reduce the dosing frequency [15]. Several recent studies have shown that Zol may be used as an anti-inflammatory drug to attenuate response of macrophages and other immune cells in several disease including arthritis, pulmonary fibrosis, breast cancer and low back pain, most often requiring repeated high dosing of Zol to realize a therapeutic effect [14, 17, 58–61]. Further earlier studies have reported that CaZol nanoparticles as used in our NiM system can reduce Zol accumulation in the bone [7, 8, 48], and increase biodistribution to non-skeletal tissue thus extending the therapeutic advantage of Zol for non-skeletal diseases.

One of the interesting findings of our study is the potent anti-inflammatory effect of CaZol NiM in suppressing NF-κB activity and reducing ROS generation. Zol has been shown to reduce the NF-κB activity in various cells especially osteoclast [62–64]. Corroborating earlier finding from literature [65], we observed a decrease in NF-κB activation in activated RAW 264.7 macrophages upon treatment with any of the three-formulation free Zol, CaZol NP and CaZol NiM-FA (Fig. 9).

Surprisingly, CaZol NiM-FA emerged as the most potent inhibitor of NF-κB activity among the three formulations, outperforming even CaZol NP, which exhibited the highest cellular uptake. (Fig. S10). Furthermore, CaZol NiM inhibited NF-κB activity within 4 h of treatment, with effects sustained at 24 h, suppressing NF-κB activity to levels similar with baseline. Since CaZol NiM-FA shows low levels but sustained release of Zol with minimal cytotoxicity, such potent anti-inflammatory response was striking. Moreover, we observed that while CaZol NiM-FA on its own does not result in activation of the NF- κB in RAW macrophages, prolonged incubation of the cells for 24 h with free Zol resulted in a low level of NF -κB activation as indicated by nuclear translocation of the phosphorylated p65 (Fig. S11). This solo effect of NF -κB activation was accompanied by a significant cell death as evident by cell number decrease (Fig. S11). These observations may be due to the detrimental effects of prolonged exposure of high Zol levels, as Zol has also been implicated to exacerbate inflammation in macrophages through several inflammatory pathways that also drive apoptosis [66, 67]. This data agrees with our cell apoptosis and viability data whereby both CaZol NP and free Zol, led to a significant decrease in cell viability as compared to CaZol NiM-FA. Altogether, these results imply that releasing Zol in a sustained manner as demonstrated herein with the CaZol NiM-FA platform may revamp Zol’s anti-inflammatory properties while minimizing its toxicity to cells.

Activation and dysregulation of NF-κB, a master pro-inflammatory regulator, plays a crucial role in mediating inflammation due to the crucial role in activation and differentiation of immune cells, including macrophages. Activation and dysregulation of NF-κB contributes to various inflammatory diseases including autoimmune disorders, arthritis and cancer. NF-κB is typically kept inactive in the cytoplasm, but various stimuli like pathogens, cytokines, and stress signals can trigger its activation, leading to its translocation to the nucleus where it binds to DNA and initiates gene transcription [68].Due to its central role in inflammation, targeting the NF-κB pathway is a promising strategy for treating cancer, inflammatory diseases, arthritis, low back pain and autoimmune diseases, and thus numerous research studies have demonstrated the inhibitory effect of Zol on NF-κB activation, highlighting its significant therapeutic potential [58, 60, 62–65]. However, these advantages of Zol are offset by the high cytotoxicity and low intracellular bioavailability due to high hydrophilicity. In fact, sustained release formulation of Zol and other bisphosphonate drugs (clodronate) in form microparticles or nanoliposomes have been used to eradicate macrophages and ameliorate inflammation by down regulation of NF-κB [56, 69]. However, complete eradication of macrophages leads to several side effects, and even accelerates disease progression in many inflammatory conditions [70, 71]. Our innovative NiM system circumvents several of these challenges in Zol delivery by serving as a depot for Zol release. The NiM system not only allows for sustained and controlled release of Zol but also maintains the NF-κB inhibitory activity without excessive cytotoxicity, enabling delivery of high Zol doses to control inflammation. Further, coupled with pH sensitive release it minimizes burst release and facilitates high degree of intracellular Zol release thus, reducing off-target effects.

Further, we also confirmed the anti-inflammatory effect of CaZol NiM by its effect on reducing ROS activation in macrophages (Fig. 10). A few studies show that Zol exhibit apoptotic effect by activation of ROS in several cell types including osteoclast and cancer cells [57, 72–74]. While activation of ROS may be required in these pathologies to restore homeostasis and kill the diseased cells, in many other inflammatory diseases, generation of ROS contributes to disease pathology and tissue damage [14, 15, 75, 76]. Our NiM platform, due to the ability to maintain high cell viability and inhibit ROS activation, expands the therapeutic utility of Zol for treating inflammatory conditions of skeletal and non-skeletal tissue marked by high ROS generation while minimizing cell and tissue damage.

Interestingly, our results indicate that the sustained release of Zol from NiM inhibits adoption of M1 polarization in macrophages (Fig. 11), further corroborating the anti-inflammatory effect of NiM. While Zol is commonly used to target and ablate tumor-associated macrophages, particularly those of the M2 phenotype, and is known to repolarize macrophages to the M1 state [15, 77], our findings reveal a previously unknown anti-inflammatory effect of Zol in inhibiting macrophage polarization towards M1 state, an immunomodulatory effect likely possible due to the sustained release of Zol from NiM.

We observe suppression of M1 phenotype (indicated by reduced CD86^+^ cells) in macrophages when treated with CaZol NiM pre- and post- activation with LPS and IFN-γ. Moreover, a significant fraction of these macrophages reverts to Mф steady state. Mф are tissue-resident macrophages which play a significant role in tissue homeostasis and immunity against infection [78]. Interestingly, a minor yet significant fraction of cells exhibited a shift toward M2 polarization, as indicated by an increase in CD206^+^ cells and (CD86^+^/206^+^) double positive cells (Fig. 11). These results align with previous reports investigating the immunomodulatory effects of Zol in suppressing dendritic cell activation [77, 78]. Although this observed expression of M2-like phenotype depicted by CD86^+^CD206^+^ was modest (~ 5%), it was statistically significant and biologically meaningful, considering that zoledronic acid (Zol) is a small molecule with indirect immunomodulatory effects (reduction in NF-κB and ROS). Notably, our ongoing (unpublished) studies demonstrate that NiM treatment achieves a level of macrophage repolarization comparable to that induced by IL-4, a potent cytokine commonly used to promote M2 polarization, further supporting the functional relevance of this shift.

Collectively our data demonstrates the potential of sustained release of Zol from multifunctional CaZol NiM for macrophage repolarization. Considering Zol is a clinically approved hydrophilic small molecule drug, the potential to repolarize macrophages without eliminating them can considerably expand its clinical utility in treating several chronic inflammatory conditions, where potent macrophage repolarization inducing cytokines when administered exogenously exert pleiotropic effects and exhibit several side-effects.

## Conclusion

In summary, we report for the first time the development of a multifunctional drug delivery system comprising calcium zoledronic acid nanoparticles embedded within microparticles (CaZol NiM) for the delivery of Zol, a potent bisphosphonate drug often limited by poor pharmacokinetics. The NiM system significantly enhanced the potency of Zol, enabling its effective application for targeted macrophage delivery and immunomodulation.

Notably, CaZol NiM-FA reduced Zol cytotoxicity, exhibited high macrophage-targeting efficiency, and attenuated macrophage inflammatory activity by downregulating NF-κB expression, suppressing ROS production, and repolarizing macrophages toward a non-inflammatory state. Unlike free Zol and CaZol NP, the NiM formulation provided a pH-responsive and sustained release profile under both acidic and physiological conditions, resulting in improved cellular viability. This controlled release behavior highlights the promise of NiM for both local and systemic delivery, minimizing premature Zol release during circulation and thereby reducing off-target toxicity.

A key advantage of this study is the demonstration of the robustness, biocompatibility, multifunctionality, and immune response modulating performance of the CaZol NiM-FA platform. However, a limitation of the current work is that the findings were validated only in a murine macrophage cell line. Confirmatory studies using human blood-derived macrophages will be critical to further advance the clinical translation of this technology. Although in vivo evaluation was beyond the scope of this initial study, the demonstrated in vitro efficacy strongly supports the potential for future preclinical investigations in animal models of degenerative and macrophage-driven inflammatory diseases, including osteoarthritis, osteoporosis, and extra-skeletal disorders. Our ongoing studies in a murine model of osteoarthritis will provide critical insights into the in vivo immunomodulatory potential of CaZol NiM-FA.

Furthermore, the versatility of this innovative delivery platform could be extended to improve the therapeutic efficacy of other biologics and small-molecule drugs that similarly suffer from poor pharmacokinetics and biodistribution. Together, these findings underscore the broad translational promise of the CaZol NiM-FA system while identifying important next steps to fully realize its clinical impact.

## Supplementary Information

Below is the link to the electronic supplementary material.

Supplementary file1 (DOCX 5.46 MB)

## Data Availability

The datasets generated and/or used during this study are available from the corresponding author upon reasonable request.
