# Supplementary material for: Bisphosphonates loaded nanoparticles in microparticles: a potential macrophage targeting and repolarizing drug delivery system
Source: Drug Deliv Transl Res. 2025 Jun 6;16(1):140–61. doi: 10.1007/s13346-025-01889-7 (PMC12682720; doi:10.1007/s13346-025-01889-7)
Supplement: Supplementary file 1 — Supplementary file1 (DOCX 5.46 MB) [file 13346_2025_1889_MOESM1_ESM.docx]

**Supplementary Information**

**Bisphosphonates Loaded Nanoparticles in Microparticles: A Potential Macrophage Targeting Immunomodulatory Drug Delivery System**

Paul N. K Sagoe^1, 2,^ Benjamin Zink^3^ and Era Jain*^1, 2^

1 Department of Biomedical and Chemical Engineering, Syracuse University, Syracuse, NY, 13244, USA

2 Bioinspired Syracuse: Institute for Material and Living System, Syracuse University, Syracuse, NY 13244, USA

3 SUNY Upstate Medical University, Syracuse, NY, 13210, USA

[pnsagoe@syr.edu](mailto:pnsagoe@syr.edu)

[zinkb@upstate.edu](mailto:zinkb@upstate.edu)

[erjain@syr.edu](mailto:erjain@syr.edu)

***Corresponding Author**

Era Jain (Ph.D.)

Biomedical and Chemical Engineering

Bioinspired Syracuse: Institute for Material and Living System

Syracuse University

Syracuse, NY, USA, 13244

Tel: 315.443.4050

Email: erjain@syr.edu

**
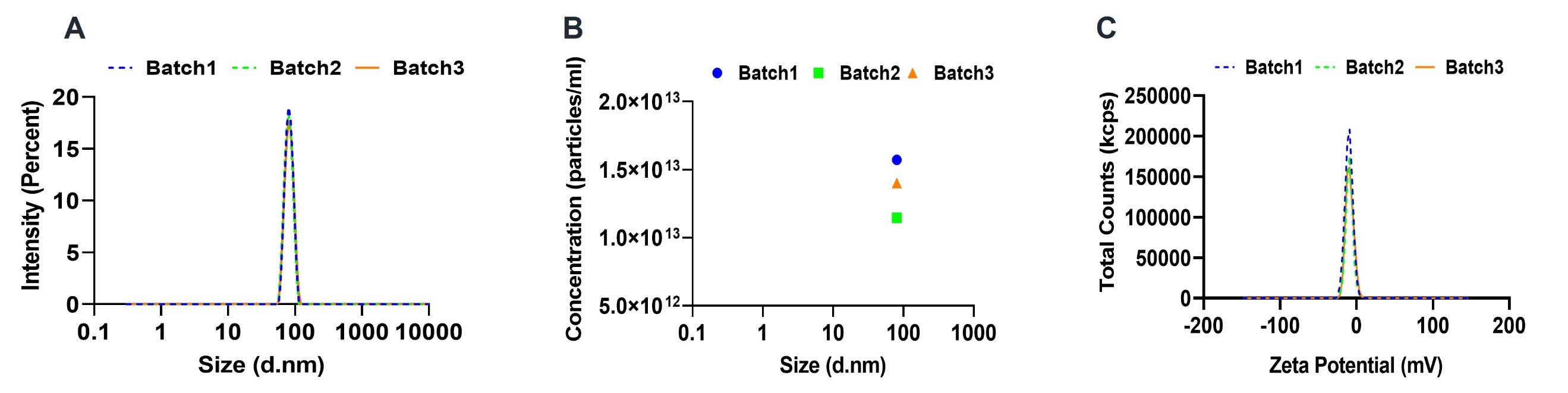
**

**Figure S1**: DLS characterization of CaZol NP prepared in three different batches. **A)** size distribution, **B)** particle concentration, **C)** zeta potential measurement. N=3 for all experiments.

**
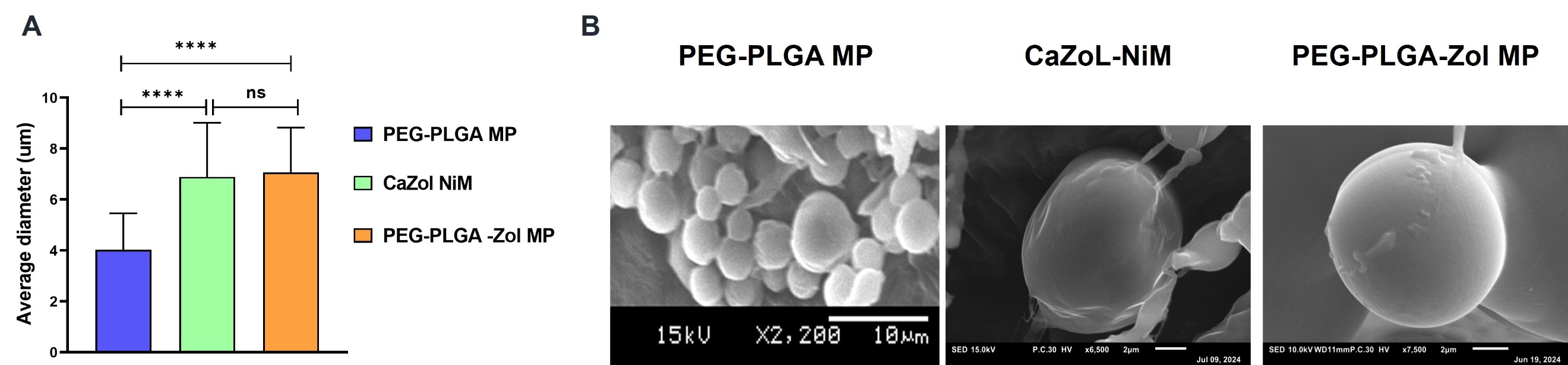
**

**Figure S2**: Effect of CaZol NP encapsulation on PEG-PLGA MP. **A)** Comparisons of average diameter of the three microparticle formulations: CaZol-NiM, Zol loaded PEG-PLGA MP and PEG-PLGA MP (blank). **B)** SEM images showing surface morphology of the three microparticle formulation. All groups were compared by one-way ANOVA with Tukey multiple comparison test. Asterisk indicates *****p* < 0.0001; ns: no statistically significant difference.

**Table S1:** Comparison between CaZol NiM and PEG-PLGA -Zol MP

| **Formulation** | **D_n_ (mean ± SD)** | **CV** | **Encapsulation Efficiency (EE%)** | **Drug Loading**  **(DL%)** |
| --- | --- | --- | --- | --- |
| CaZol NiM | 6.9 ± 2.1 um | 0.30 | 55.8 ± 9.0 | 3.9 ± 0.1 |
| PEG-PLGA-Zol MP | 7.1 ± 1.8 um | 0.25 | 25.9 ± 8.7 | 4.2 ± 0.9 |

**
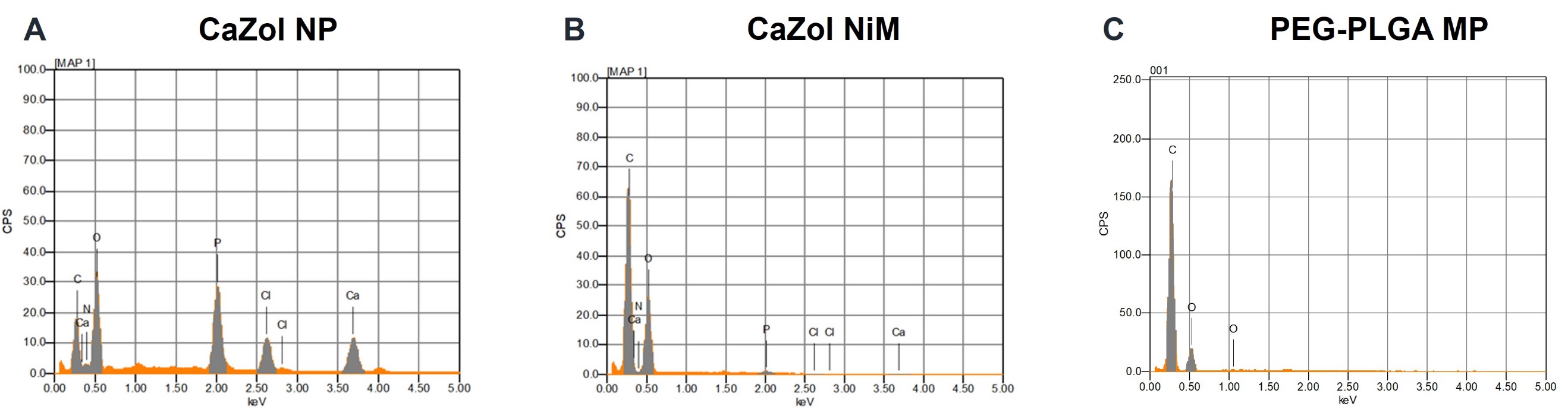
**

**Figure S3**: EDAX Analysis of: A) CaZol NP, B) CaZol NiM, and C) PEG-PLGA MP (blank).

Elemental composition of Plot A and B confirms the detection of Phosphorus (P), Calcium (Ca), Chlorine (Cl) and Nitrogen (N), all indicative of the presence of CaZol nanoparticles. These elements were absent in Plot C, which represents the control, PEG-PLGA MP (blank).

**
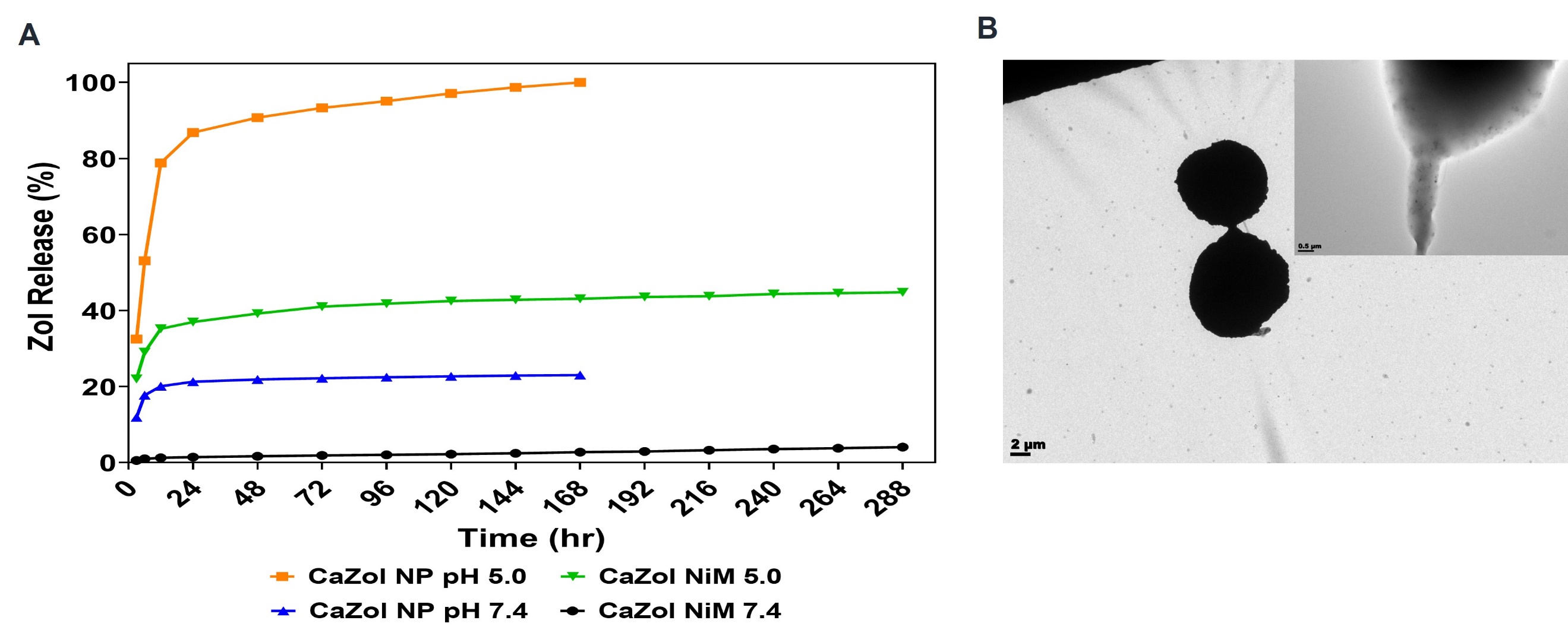
**

**Figure S4**: In-vitro release of Zol from CaZol-NiM and CaZol NP. **A)** Release kinetics of Zol from CaZol NiM and CaZol NP at pH 7.4 and 5.0 as measured by ICP-OES. (N=1)

**B)** TEM image of CaZol NP being released from CaZol NiM in a release medium conditioned at pH 7.4 PBS in a 37C shaker incubator at 7 days.

**
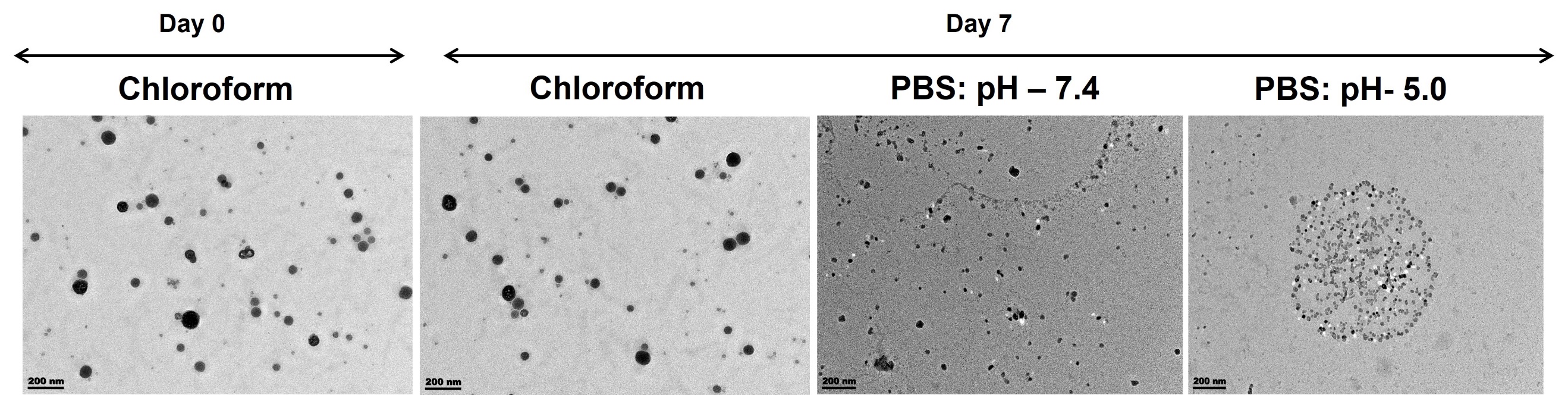
**

**Figure S5**: Effect of pH on CaZol NP stability at room temperature in chloroform and PBS at pH 7.4 and pH 5.0 after 7 days.

**
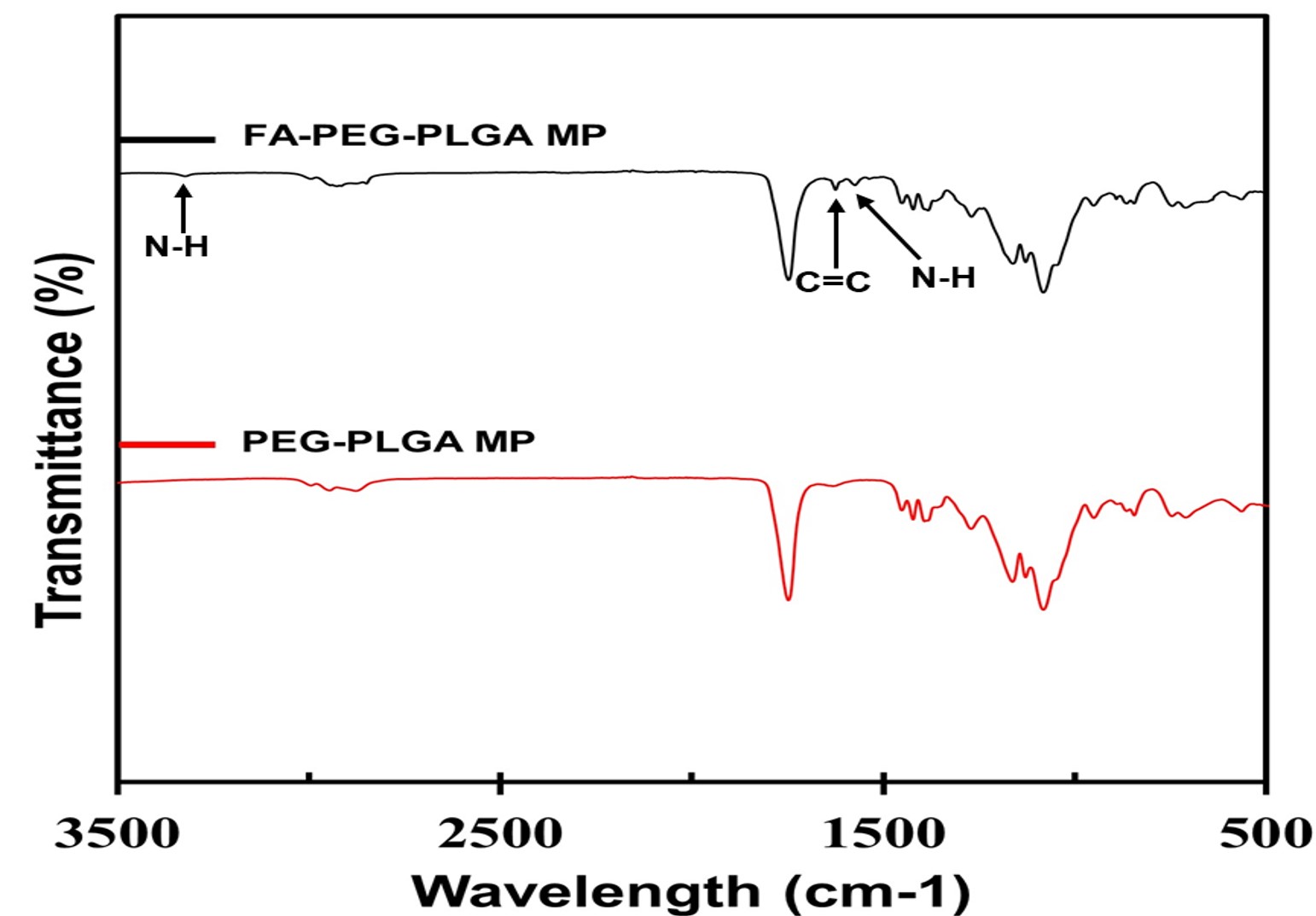
**

**Figure S6**: FTIR spectrum confirming successful incorporation of FA on PEG-PLGA MP

The presence of FA on PEG-PLGA microparticles was confirmed by FTIR analysis, with characteristic peaks observed at 1574 cm⁻¹ (N-H bending), 1625 cm⁻¹ (C=C bending), and 3423 cm⁻¹ (N-H amine stretching), indicating the successful incorporation of FA as a ligand. These peaks were not observed in the PEG-PLGA MP synthesized without folic acid conjugated PEG-PLGA polymer.


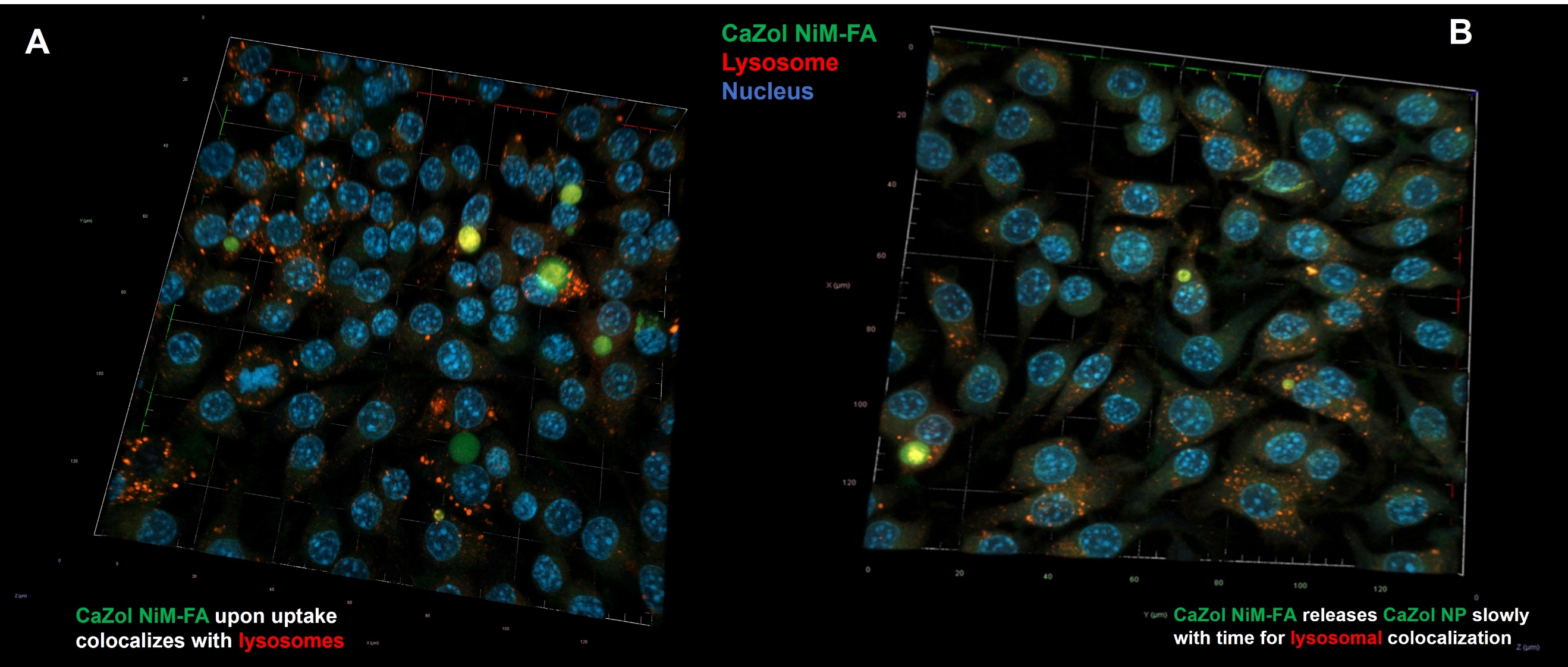


**Figure S7**: 3D representation of the Intracellular localization of coumarin-6 stained CaZol particles with lysosome after incubating with CaZol NiM-FA for A) 2hrs and B) 4hrs.

Confocal images of RAW macrophages when incubated with CaZol NiM-FA and immunostained with lysotracker dye. With time, CaZol NiM-FA effectively and slowly releases CaZol NP allowing for their colocalization with lysosome, indicated by orange coloration from the merging of green stained coumarin-6 particles and red stained lysosomal compartments within cell. The scale bar represented is of 20µm margin for X, Y, Z orientation.


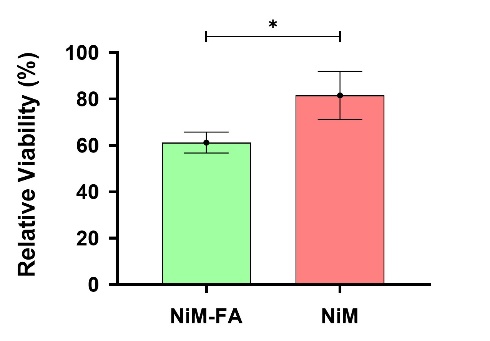


**Figure S8**: Assessment of cytotoxicity of non-targeted CaZol NiM and targeted CaZol NiM-FA. Cells were treated for 24 hours with NiM and NiM-FA at equivalent Zol concentration (25 ug/ml) and their toxicity was confirmed via MTT assay. Statistical analysis was computed with t-test and the significance between the two treatment conditions was determined at a 95% confidence level with the threshold: *p*< 0.05 (*)

**
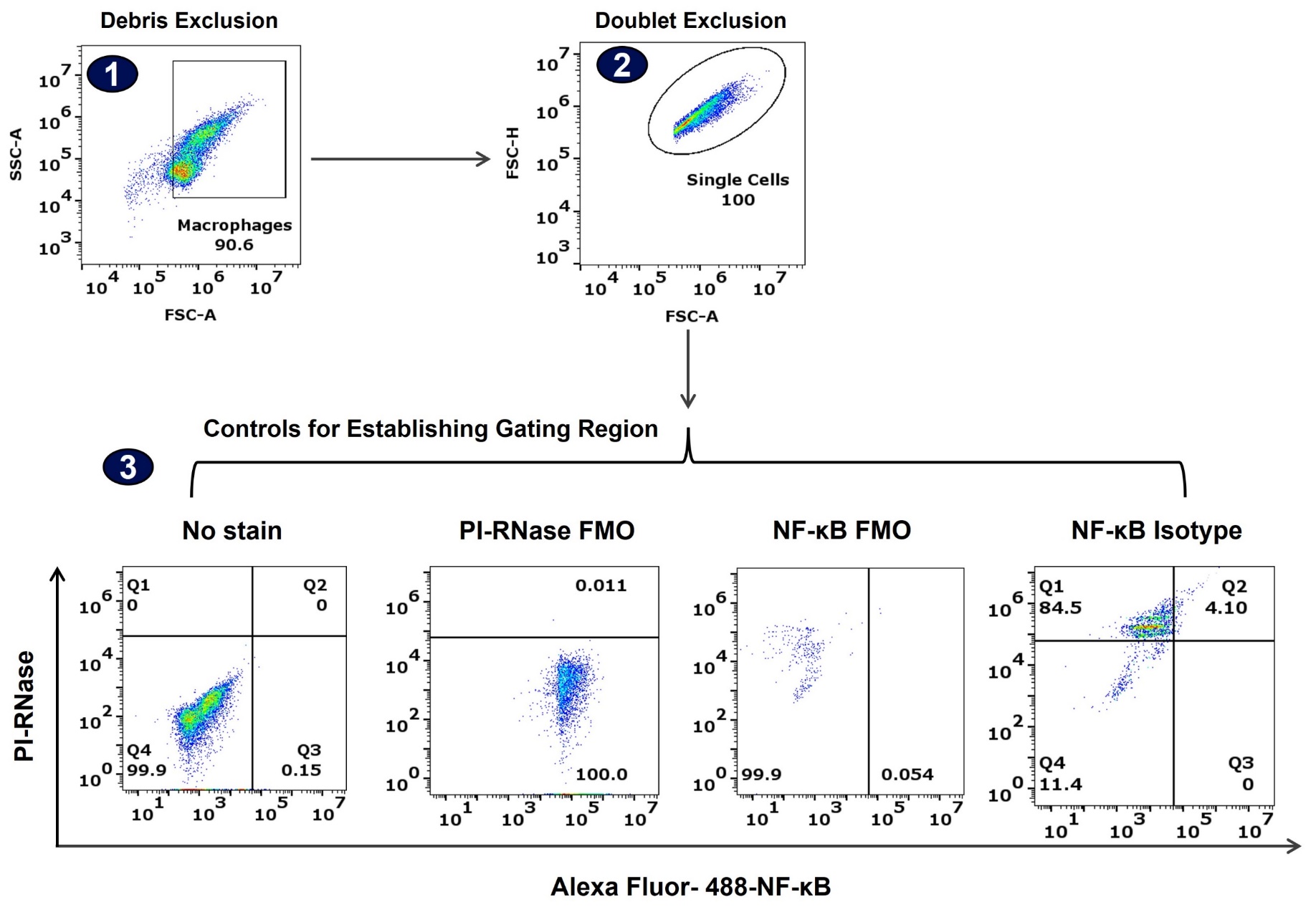
**

**Figure S9**: Gating Strategy for Detecting nuclear translocation due to NF-κB activation. Positive regions of the gated quadrants were defined by fluorescence minus one (FMO) control, NF-κB FMO and PI-RNase FMO.


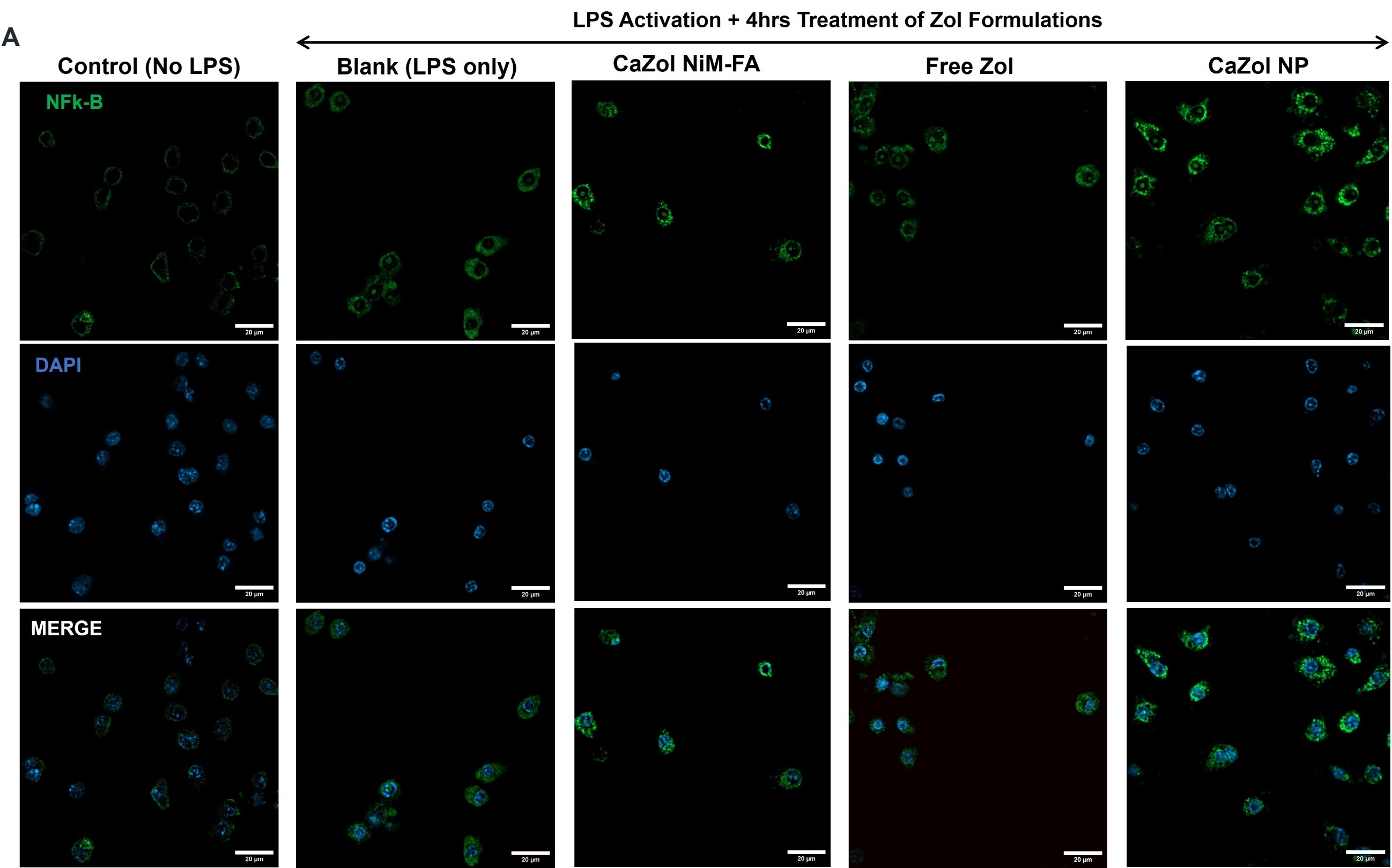


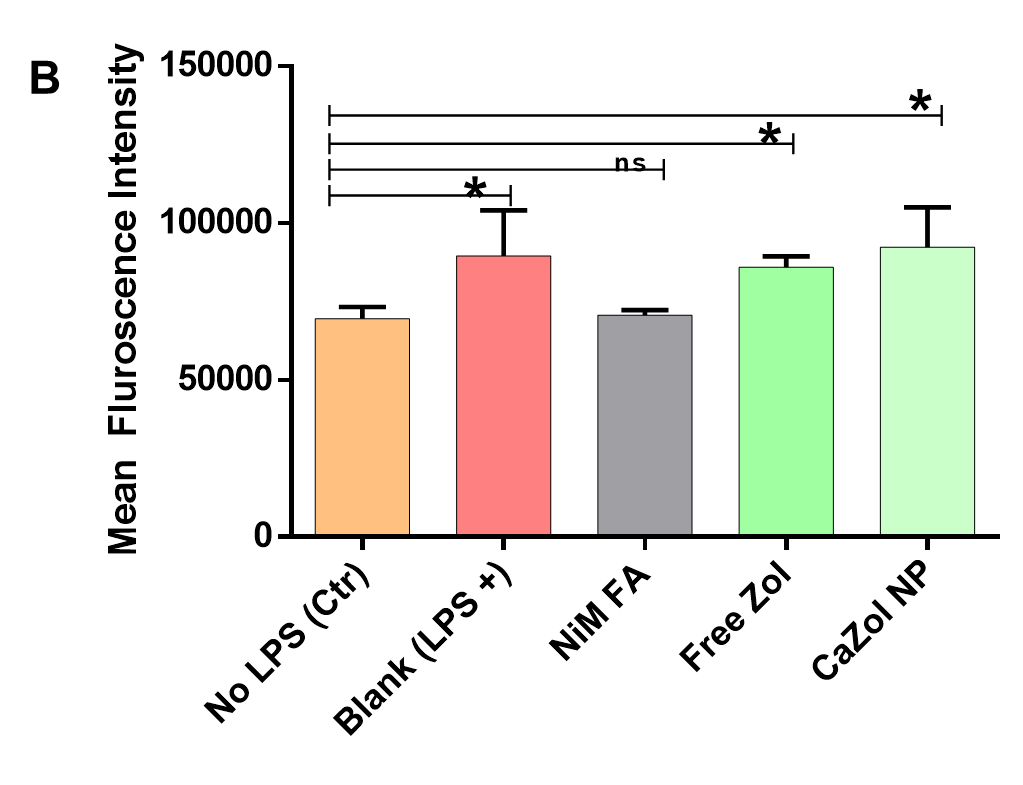


**Figure S10:** Effects of various Zol formulations on NF-κB activity. **A)** Confocal images of immunostained cells depicting intracellular location of NF-κB p65 in activated macrophages treated with various Zol formulations for 4 h. **B)** Quantification of nuclear translocation and activation of NF-κB by flow cytometry and comparison of the MFI values of cells populations double positive for PI/RNase and Alexa Fluor 488-NF-κB amongst different groups. Note each Zol formulation contained a dose equivalent of 25 μg/ml of Zol and activated macrophages were treated for 4 h with each formulation. Multiple groups were compared using one-way ANOVA with Bonferroni multiple comparison test. Asterisk indicates the following thresholds: **p* < 0.05.

**
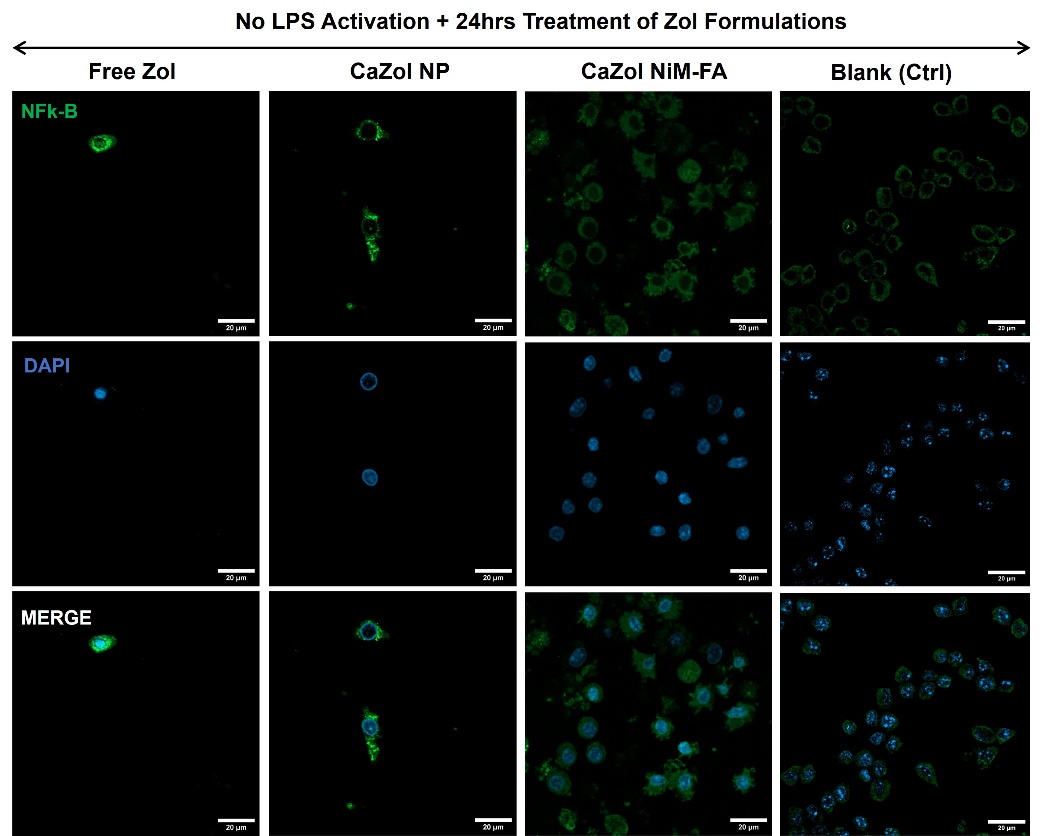
**

**Figure S11**: Effects of Zol on NF-κB activity in non-activated macrophages. Confocal microscopy Immunofluorescence staining of non-LPS activated RAW 264.7 macrophage cells incubated with free Zol, CaZol NP, CaZol NiM-FA for 24 hours.


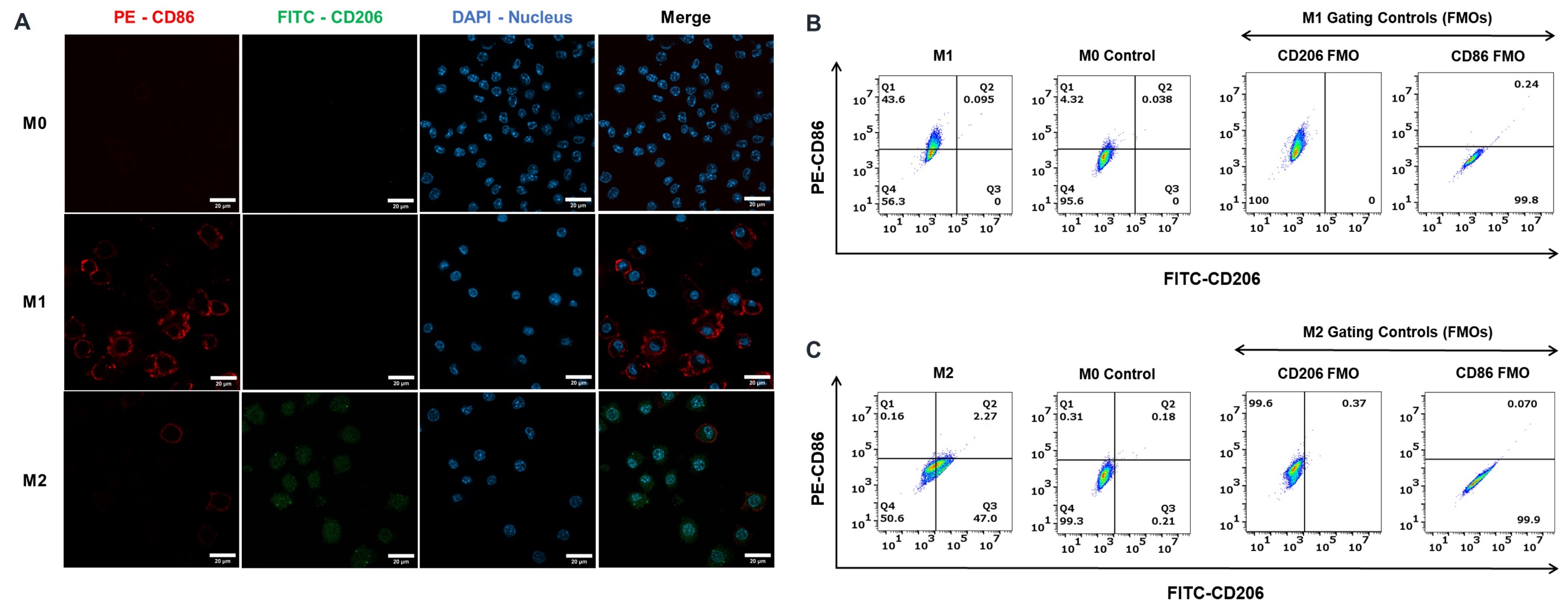


**Figure S 12:** Assessment of cytokine induced macrophage polarization state. **A)** Confocal images of macrophages in different states of polarization and expressing identifying surface makers. LPS and IFN-γ activated macrophages depicted as M1 polarized macrophages show the expression of CD86 while IL-4 and IL-13 activated macrophages also depicted as M2 polarized macrophages show CD206 expression. **B, C)** M1 and M2 flow cytograms quantifying the expression of the surface markers in cytokine induced macrophage polarization. Confirming the results from immunostained confocal microscopy images, M1 macrophages showing high expression of CD86 in LPS and IFN-γ activated M1 and while IL-4 and IL-13 activated M2 macrophages show CD206 surface marker expression. Mф macrophages minimally or do not express any of these surface markers.


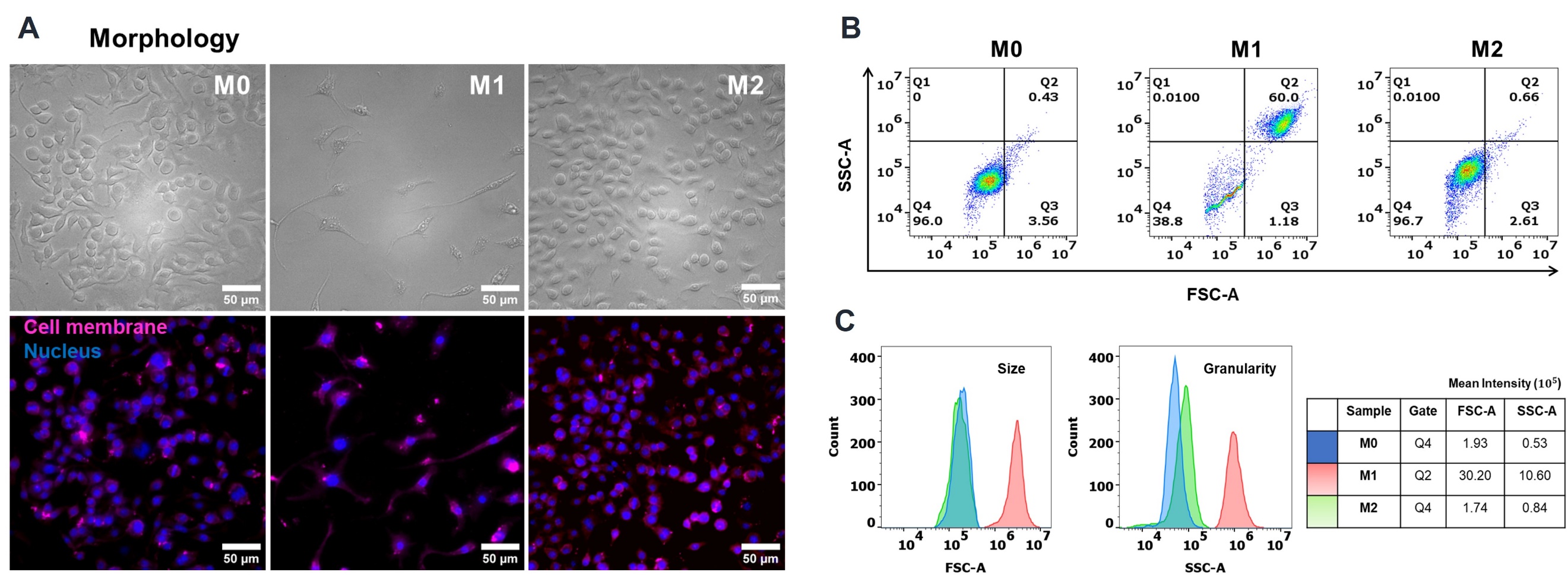


**Figure S 13:** Assessment of cytokine induced macrophage polarization state. **A)** Fluorescent and brightfield images showing change in macrophage morphology indicative of their polarization state. M1 macrophages show spindle-like and highly spread cell morphology while Mф and M2 macrophages have relatively round morphology with few and short cell membrane protrusions. **B, C)**. Flow cytogram and histogram show FSC vs SSC plot estimating granularity and size of macrophages in different polarization states. Corroborating the results obtained from brightfield images, M1 macrophages show the largest size and highest granularity as indicated by the right shift on Count vs FSC plot and Count vs SSC plot respectively. Notably, Mф and M2 macrophages were observed to be similar in size, with M2 having greater granularity.


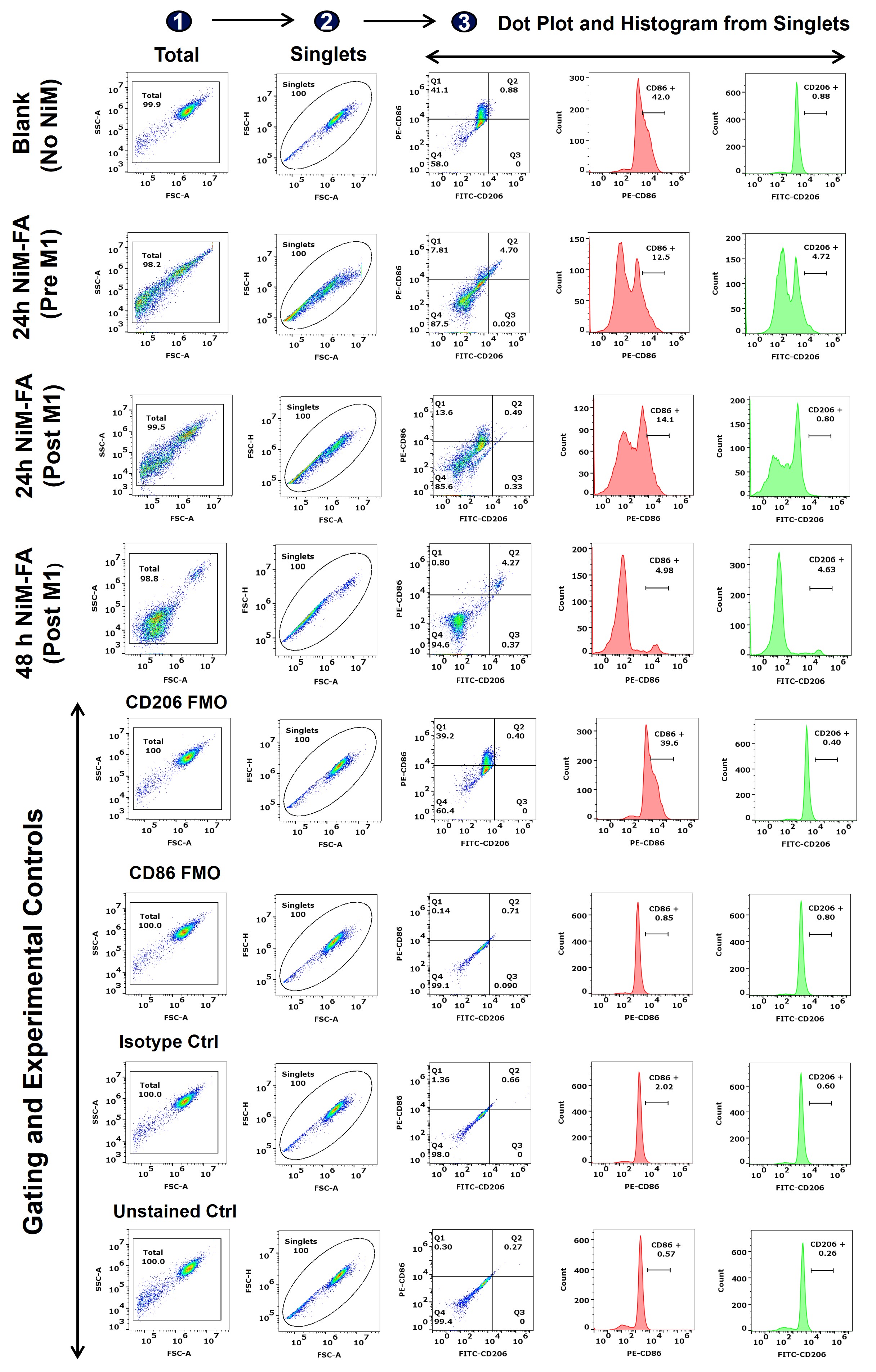


**Figure S14**: Gating Strategy for evaluating macrophage phenotype repolarization.

Flow cytometry plots show expression of CD86 (M1 marker) and CD206 (M2 marker) by M1 polarized cells (LPS + IFN-γ) with or without CaZol-NiM-FA treatment for 24h or 48h (Pre-M1 and Post M1 activation). Gating regions were determined by appropriate FMO controls with a slight positive offset of 0.40% for CD 206 FMO and 0.85% for CD 86 FMO over the gated boundary.
